# Optimized testing strategy for the diagnosis of GAA-*FGF14* ataxia/spinocerebellar ataxia 27B

**DOI:** 10.1038/s41598-023-36654-8

**Published:** 2023-06-15

**Authors:** Céline Bonnet, David Pellerin, Virginie Roth, Guillemette Clément, Marion Wandzel, Laëtitia Lambert, Solène Frismand, Marian Douarinou, Anais Grosset, Ines Bekkour, Frédéric Weber, Florent Girardier, Clément Robin, Stéphanie Cacciatore, Myriam Bronner, Carine Pourié, Natacha Dreumont, Salomé Puisieux, Pablo Iruzubieta, Marie-Josée Dicaire, François Evoy, Marie-France Rioux, Armand Hocquel, Roberta La Piana, Matthis Synofzik, Henry Houlden, Matt C. Danzi, Stephan Zuchner, Bernard Brais, Mathilde Renaud

**Affiliations:** 1 Laboratoire de Génétique Médicale, Hôpitaux de Brabois - CHRU de Nancy, Nancy, France; 2 INSERM-U1256 NGERE, Université de Lorraine, Nancy, France; 3 Department of Neuromuscular Diseases, UCL Queen Square Institute of Neurology and The National Hospital for Neurology and Neurosurgery, University College London, London, UK; 4 Department of Neurology and Neurosurgery, Montreal Neurological Hospital and Institute, McGill University, Montreal, QC Canada; 5 Service de Neurologie, CHRU de Nancy, Nancy, France; 6 Service de Génétique Clinique, Hôpitaux de Brabois - CHRU de Nancy, Nancy, France; 7 Department of Neurology, Donostia University Hospital, San Sebastian, Spain; 8 Neuroscience Area, Biodonostia Health Research Institute, San Sebastian, Spain; 9 Network Center for Biomedical Research in Neurodegenerative Diseases (CIBERNED), Madrid, Spain; 10 Faculty of Medicine and Health Sciences, Université de Sherbrooke, Sherbrooke, QC Canada; 11 Department of Diagnostic Radiology, McGill University, Montreal, QC Canada; 12 Department of Neurodegenerative Diseases, Hertie-Institute for Clinical Brain Research and Center of Neurology, University of Tübingen, Tübingen, Germany; 13 German Center for Neurodegenerative Diseases (DZNE), Tübingen, Germany; 14 Dr. John T. Macdonald Foundation Department of Human Genetics and John P. Hussman Institute for Human Genomics, University of Miami Miller School of Medicine, Miami, FL USA; 15 Department of Human Genetics, McGill University, Montreal, QC Canada

**Keywords:** Clinical genetics, Neurological disorders

## Abstract

Dominantly inherited GAA repeat expansions in *FGF14* are a common cause of spinocerebellar ataxia (GAA-*FGF14* ataxia; spinocerebellar ataxia 27B). Molecular confirmation of *FGF14* GAA repeat expansions has thus far mostly relied on long-read sequencing, a technology that is not yet widely available in clinical laboratories. We developed and validated a strategy to detect *FGF14* GAA repeat expansions using long-range PCR, bidirectional repeat-primed PCRs, and Sanger sequencing. We compared this strategy to targeted nanopore sequencing in a cohort of 22 French Canadian patients and next validated it in a cohort of 53 French index patients with unsolved ataxia. Method comparison showed that capillary electrophoresis of long-range PCR amplification products significantly underestimated expansion sizes compared to nanopore sequencing (slope, 0.87 [95% CI, 0.81 to 0.93]; intercept, 14.58 [95% CI, − 2.48 to 31.12]) and gel electrophoresis (slope, 0.84 [95% CI, 0.78 to 0.97]; intercept, 21.34 [95% CI, − 27.66 to 40.22]). The latter techniques yielded similar size estimates. Following calibration with internal controls, expansion size estimates were similar between capillary electrophoresis and nanopore sequencing (slope: 0.98 [95% CI, 0.92 to 1.04]; intercept: 10.62 [95% CI, − 7.49 to 27.71]), and gel electrophoresis (slope: 0.94 [95% CI, 0.88 to 1.09]; intercept: 18.81 [95% CI, − 41.93 to 39.15]). Diagnosis was accurately confirmed for all 22 French Canadian patients using this strategy. We also identified 9 French patients (9/53; 17%) and 2 of their relatives who carried an *FGF14* (GAA)_≥250_ expansion. This novel strategy reliably detected and sized *FGF14* GAA expansions, and compared favorably to long-read sequencing.

## Introduction

Late-onset cerebellar ataxias (LOCAs) are a group of neurodegenerative conditions that have until recently largely challenged molecular diagnosis^1, 2^. Despite recent advances in our understanding of the genetic basis of these conditions, a genetic diagnosis is reached in less than 50% of patients with LOCA^3, 4^. Dominantly inherited GAA repeat expansions in the first intron of the fibroblast growth factor 14 gene (*FGF14*) have recently been reported as a common cause of LOCA (GAA-*FGF14* ataxia; spinocerebellar ataxia 27B [MIM: 620174]), accounting for 10 to 61% of unsolved cases in various cohorts^5, 6^. Current data support a pathogenic threshold of (GAA)_≥250_ repeat units. Core clinical features of GAA-*FGF14* ataxia include slowly progressive cerebellar ataxia, early episodic symptoms, downbeat nystagmus, diplopia, and dizziness/vertigo^5^. Molecular confirmation of the *FGF14* GAA repeat expansion has thus far mostly relied on long-read sequencing, a technology that is not yet widely available in clinical diagnostic laboratories. Given the high reported frequency of GAA-*FGF14* ataxia, there is an immediate need to establish a standardized, accessible, and validated molecular strategy for diagnosing this novel repeat expansion disorder in clinical diagnostic laboratories.

Herein, we propose a strategy that combines long-range polymerase chain reaction (LR-PCR), bidirectional repeat-primed PCRs (RP-PCRs), and Sanger sequencing to detect and resolve *FGF14* GAA repeat expansions in clinical diagnostic settings. We further demonstrate the applicability of this diagnostic approach in a cohort of French patients with unsolved LOCA.

## Methods

### Patient enrollment

All participants provided written informed consent. This study was conducted in accordance with the Declaration of Helsinki.

#### French Canadian cohort

Patients were recruited at the Montreal Neurological Hospital of the McGill University Health Centre (Montreal, QC, Canada). The institutional review board of the McGill University Health Centre approved this study (MPE-CUSM-15-915). The French Canadian participants included 22 patients with GAA-*FGF14* ataxia and six controls that were reported previously^5^. All participants underwent genotyping of the *FGF14* repeat locus by agarose gel electrophoresis of LR-PCR amplification products and targeted long-read nanopore sequencing, as described previously^5^.

#### French cohort

Fifty-three index patients with LOCA and two affected relatives were recruited at the Centre Hospitalier Régional Universitaire de Nancy (France). The institutional review board of the Centre Hospitalier Régional Universitaire de Nancy approved this study (2020PI220). Patients were enrolled if meeting the following inclusion criteria: (a) progressive ataxia with onset at or after age 30; (b) no clinical features suggestive of multiple system atrophy (MSA); (c) exclusion of acquired causes; and (d) exclusion of known genetic causes by testing negative on an ataxia gene panel and for common coding and non-coding repeat expansion disorders. The Scale for the Assessment and Rating of Ataxia (SARA)^7^ was recorded when possible. Magnetic resonance imaging (MRI) of the brain was obtained for nine patients with GAA-*FGF14* ataxia.

### Molecular analysis (Fig. 1 and Supplementary Fig. S1)

**Figure 1:**
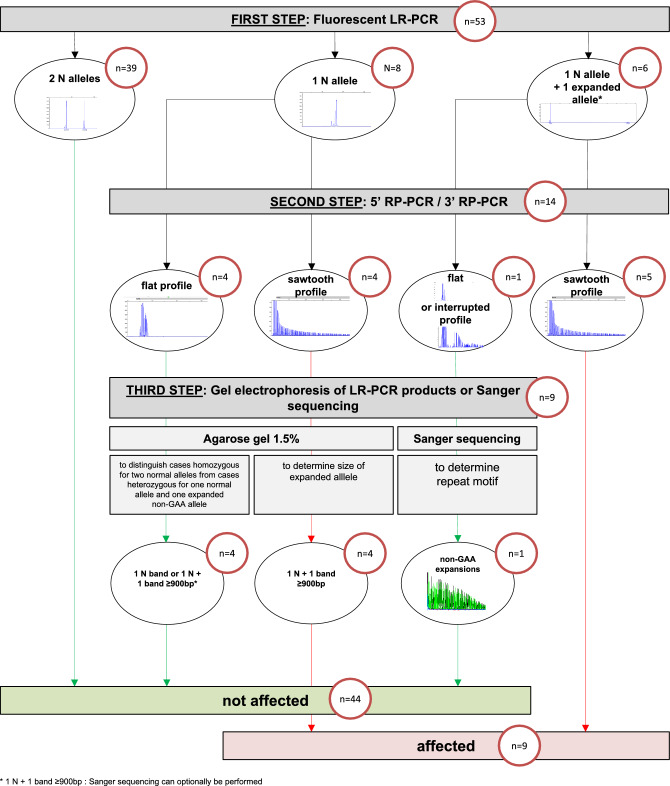
Testing strategy for the diagnosis of GAA-*FGF14* ataxia. The number of samples in the French Cohort of 53 index patients processed at each step is indicated in the red circles. Normal alleles have (GAA)_<250_ repeats and expanded alleles have (GAA)_≥250_ repeats. *Two expanded alleles are possible. N allele, normal allele < 250 repeat units; LR-PCR, long-range polymerase chain reaction; RP-PCR, repeat-primed PCR.

The primer sequences and experimental conditions are provided in Supplementary Table S1. Genomic DNA was isolated from peripheral blood using standard methods (see Supplementary Methods). DNA concentrations were measured with the NanoDrop Spectrophotometer.

#### First step: fluorescent long-range PCR

The intronic *FGF14* GAA repeat locus was amplified by fluorescent long-range PCR (fLR-PCR). fLR-PCR products were analyzed on an ABI 3130*xl*, 3500*xl*, or 3730*xl* DNA Analyzer (Applied Biosystems, Foster City, CA, USA) with a 50-cm POP-7 capillary using the GeneScan 1200 Liz Dye Size Standard (catalog no. 4379950, Applied Biosystems) following the manufacturer’s instructions (Supplementary Table S2). Results were analyzed using the GeneMapper software using the built-in microsatellite default settings (version 6.0, Applied Biosystems). The PCR primers are predicted to amplify a 300 bp fragment based on the reference sequence, which includes 50 GAA triplets. The size of the alleles (expressed in triplet repeat units) was calculated using the following formula: (size of PCR amplification product—150)/3.

#### Second step: bidirectional RP-PCRs

Two RP-PCRs targeting the 5′ end (5′ RP-PCR) and the 3′ end (3′ RP-PCR) of the locus were used in parallel to ascertain the presence of a GAA expansion at the repeat locus. RP-PCR products were analyzed on an ABI 3130*xl*, 3500*xl*, or 3730*xl* DNA Analyzer with a 50-cm POP-7 capillary using the GeneScan 1200 Liz Dye Size Standard following the manufacturer’s instructions (Supplementary Table S2). Results were analyzed using the GeneMapper software using the built-in microsatellite default settings. The presence of characteristic saw-toothed products indicated the presence of a GAA repeat expansion at the *FGF14* repeat locus.

#### Third step: gel electrophoresis of long-range PCR products and Sanger sequencing

1. Samples with a single normal allele detected on fLR-PCR

Cases with a single normal allele detected by capillary electrophoresis of fLR-PCR products, regardless of their RP-PCR profiles, next underwent gel electrophoresis of LR-PCR amplification products using a 1.5% agarose gel. This step allows for distinguishing cases homozygous for two normal alleles from cases heterozygous for one normal allele and one expanded allele that is too large (> 400 repeat units, see Results) to be detected by capillary electrophoresis. The motif of any expansion identified at this stage is determined by the RP-PCR profiles generated during step 2, with sawtooth profiles indicating GAA expansions and flat profiles indicating non-GAA expansions. Amplification products were migrated for 3h30 at 150 V and 300 mA. Agarose gels were imaged with the Uvidoc HD6 Gel Documentation System (Uvitec) using the default UV-Gel settings in the Uvitec-1D software. The molecular weight of PCR amplification products was measured by the molecular weight detection tool in the Uvitec-1D software (Uvitec).

2. Samples with at least one allele of 250 or more repeat units detected on fLR-PCR and no sawtooth profile or interrupted profile on RP-PCRs.

Cases with at least one allele of 250 or more repeat units and no sawtooth profile or an interrupted profile on RP-PCRs next underwent Sanger sequencing to determine the sequence and repeat motif of the *FGF14* locus. Sanger sequencing of LR-PCR amplification products using the BigDye Terminator v1.1 Sequencing kit (catalog no. 4337451, Applied Biosystems) was performed using the ABI 3130*xl* DNA Analyzer (Supplementary Table S3). The resulting sequences were analyzed using Sequence Scanner version 1.0 software (Applied Biosystems) or SnapGene v.5.0.8 software (Dotmatics).

The performance of the PCR protocols was assessed using different formulations of Taq DNA polymerase, different input amounts of genomic DNA, and genomic DNA extracted from saliva, formalin-fixed paraffin-embedded cerebral tissue, and fresh frozen cerebral tissue (see Supplementary Methods and Supplementary Results). We further assessed the effect of DNA shearing on the results of the proposed strategy (see Supplementary Methods and Supplementary Results).

### Statistical analysis

We compared methods against each other using the Passing-Bablok regression model (R package: mcr using the function *mcreg(dataset1, dataset2, method.reg* = *“PaBa”)*). Confidence interval of the slope that does not include 1 indicates statistically significant evidence of a proportional bias between two methods. Confidence interval of the intercept that does not include 0 indicates statistically significant evidence of a systematic bias between two methods. Linear relationship between two sets of measurements was tested by the CUSUM test of linearity. We used the Bland–Altman analysis to compare the measurements of the same variable by two methods (GraphPad Prism 9). Correlations were calculated using the Pearson correlation coefficient. We analyzed the data in R (version 4.1) and GraphPad Prism 9. *P* value of < 0.05 was considered significant. All analyses were two-tailed.

## Results

### *FGF14* repeat sizing by fLR-PCR

We first determined how repeat size estimates of larger alleles by fLR-PCR compare and correlate to (1) targeted long-read nanopore sequencing and (2) LR-PCR estimates by agarose gel electrophoresis. We used 28 French Canadian participants for whom long-read sequencing data was available and measured repeat length by fLR-PCR. Nanopore sizing was performed on size-selected alleles of more than 80 repeat units to increase coverage depth, as described previously^5^.

**Figure 2:**
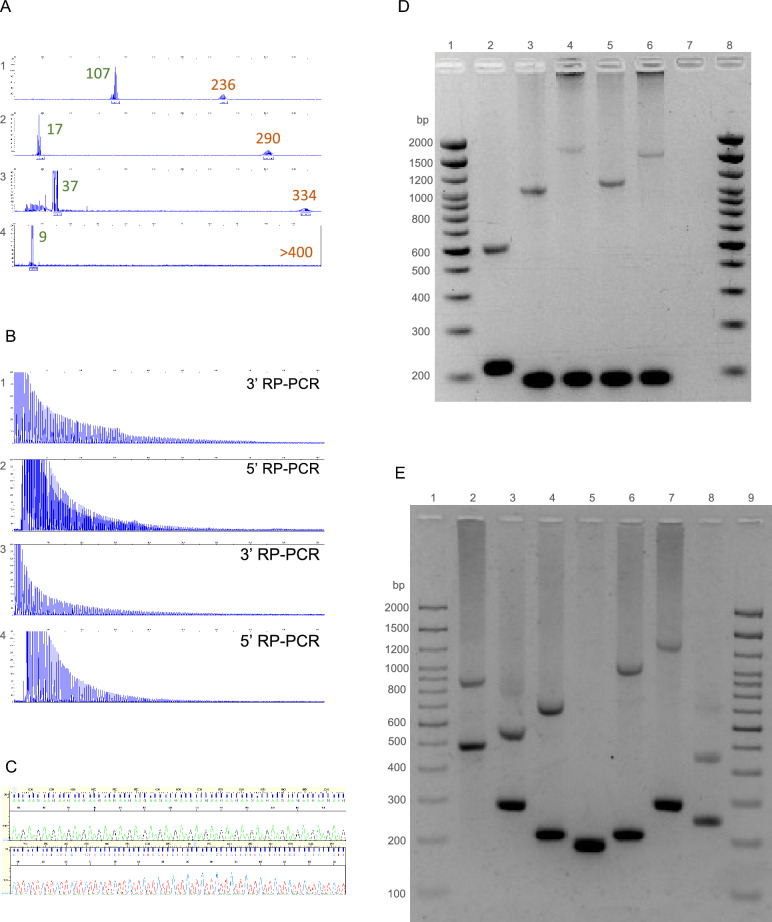
Molecular analysis of the *FGF14* repeat locus. (**A**) Fluorescent long-range PCR of the *FGF14* repeat locus of four patients with late-onset cerebellar ataxia. The calculated number of repeat units of each allele (before correction) is indicated for each of the four patients. (**B**) 5′ RP-PCR and 3′ RP-PCR of the *FGF14* repeat locus of two patients carrying a (GAA)_≥250_ repeat expansion in *FGF14*. (**C**) Sanger sequencing of a patient carrying a (GAA)_≥250_ repeat expansion showing GAA repeats: (GAA)_n_ on forward strand and (TTC)_n_ on reverse strand. (**D**) Agarose gel electrophoresis (1.5%), lanes 1 and 8: 2,000 bp molecular weight marker, 2: 17/132 repeat units, 3: 9/284 repeat units, 4: 9/510 repeat units, 5: 9/313 repeat units, 6: 9/467 repeat units, and 7: negative control. (**E**) Agarose gel electrophoresis (1.5%), lanes 1 and 9: 2,000 bp molecular weight marker, 2: 107/236 repeat units, 3: 40/132 repeat units, 4: 16/182 repeat units, 5: 8/9 repeat units, 6: 17/319 repeat units, 7: 43/370 repeat units, and 8: 28/94 repeat units. Agarose gels were imaged with the Uvidoc HD6 Gel Documentation System using the default UV-Gel settings in the Uvitec-1D software, which resulted in overexposure. The gels presented in D and E were cropped for clarity of presentation. The original gels are presented in Supplementary Fig. S10.

We observed that expansions (GAA)_>400_ repeat units could not be accurately sized by fLR-PCR, as shown in Fig. 2A, as they fall beyond the limit of detection of capillary electrophoresis. Therefore, such large expansions needed to be sized by agarose gel electrophoresis of LR-PCR amplification products.

**Figure 3:**
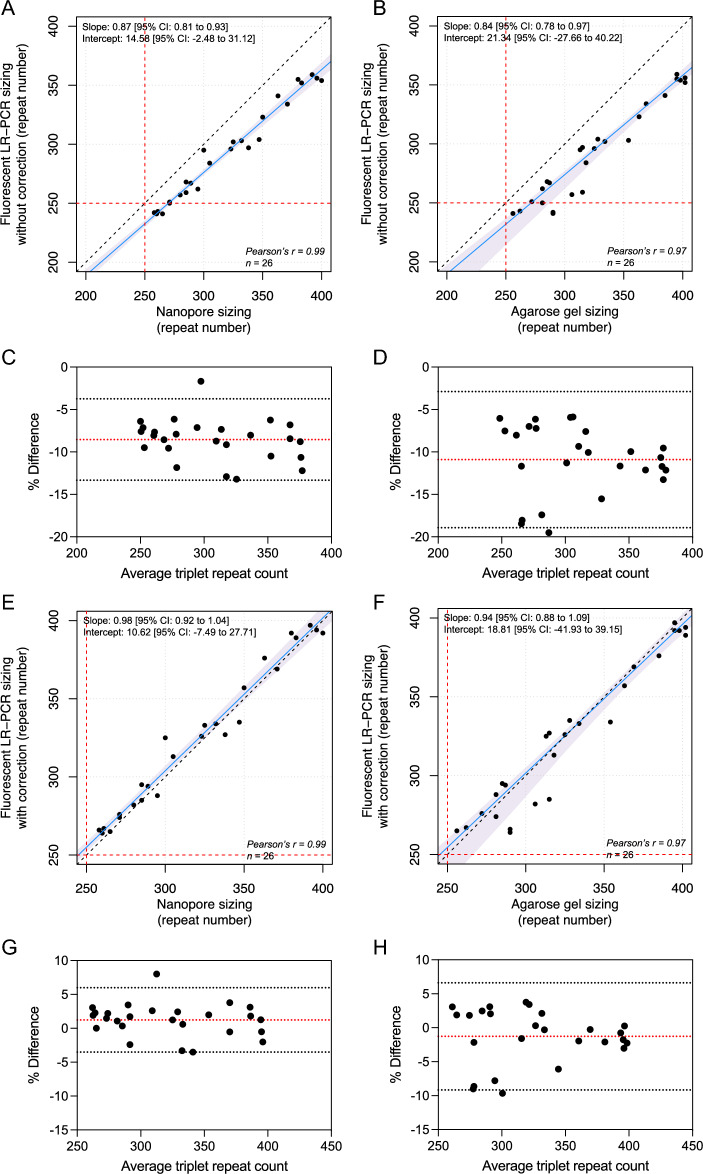
*FGF14* allele size estimates by fluorescent LR-PCR, long-read nanopore sequencing, and agarose gel electrophoresis. Passing-Bablok regression (blue lines) with 95% confidence interval (shaded blue areas) for allele size measured by (**A**) fLR-PCR and nanopore sequencing, (**B**) fLR-PCR and gel electrophoresis, (**E**) fLR-PCR (with correction) and nanopore sequencing, and (**F**) fLR-PCR (with correction) and gel electrophoresis. The dashed black lines show the identity line and the dashed red lines show the pathogenic threshold of (GAA)_≥250_ repeats. Bland–Altman plots show the percentage difference between size estimates measured by fLR-PCR and (**C**) targeted nanopore sequencing or (**D**) agarose gel electrophoresis as a function of the average of the two measurements for each sample. Plots show the percentage difference between size estimates measured by corrected fLR-PCR and (**G**) targeted nanopore sequencing or (**H**) agarose gel electrophoresis as a function of the average of the two measurements for each sample. The dashed red lines show the mean bias between two techniques and the dashed gray lines show the limits of agreement, defined as the mean percentage difference ± 1.96 SD.

Consistent with what has previously been reported with other trinucleotide repeat expansions^8–12^, we observed a significant discrepancy between sizes of larger alleles measured by capillary electrophoresis and long-read sequencing or agarose gel electrophoresis. We compared methods using the Passing–Bablok regression model^13^, and found a statistically significant proportional bias between nanopore sequencing and fLR-PCR (slope, 0.87 [95% CI, 0.81 to 0.93] and intercept, 14.58 [95% CI, − 2.48 to 31.12]) and gel electrophoresis and fLR-PCR (slope, 0.84 [95% CI, 0.78 to 0.97] and intercept, 21.34 [95% CI, − 27.66 to 40.22]) (Fig. 3A,B). The Bland–Altman analysis confirmed a negative bias of − 8.54% (95% limits of agreement, − 13.34% to − 3.75%) between fLR-PCR and nanopore sequencing, and of − 10.91% (95% limits of agreement, − 18.93 to − 2.90%) between fLR-PCR and gel electrophoresis (Fig. 3C,D). In comparison, the latter techniques yielded comparable size estimates (slope, 1.01 [95% CI, 0.90 to 1.11] and intercept, 5.35 [95% CI, − 29.98 to 44.57]) (Supplementary Fig. S9). The Bland–Altman analysis of both techniques showed a positive bias of + 2.55% (95% limits of agreement, − 6.53% to + 11.63%) (Supplementary Fig. S9). These results confirm that capillary electrophoresis systematically underestimates the size of expanded alleles compared to nanopore sequencing and gel electrophoresis, and suggest that GAA repeat-containing DNA fragments migrate faster than predicted. Faster migration of triplet repeat-containing fragments is thought to result from altered electrophoretic properties of repetition-rich DNA^11, 12^. The systematic underestimation by capillary electrophoresis resulted in a false negative result in four of 28 participants (14%) of the French Canadian cohort carrying an *FGF14* expansion. To compensate for this underestimation, we used a set of four alleles whose sizes have been established by long-read sequencing and applied the least squares method to generate a straight line that fits our data and calculated its slope and y-intercept (Supplementary Table S5). Application of this correction resulted in expansion size estimates being comparable between nanopore sequencing and fLR-PCR (slope, 0.98 [95% CI, 0.92 to 1.04] and intercept, 10.62 [95% CI, − 7.49 to 27.71]) and gel electrophoresis and fLR-PCR (slope, 0.94 [95% CI, 0.88 to 1.09] and intercept, 18.81 [95% CI, − 41.93 to 39.15]), as assessed by the Passing–Bablok regression model (Fig. 3E,F). The Bland–Altman analysis showed a positive bias of + 1.23% (95% limits of agreement, − 3.52% to + 5.98%) between corrected fLR-PCR and nanopore sequencing, and a negative bias of − 1.27% (95% limits of agreement, − 9.16% to + 6.61%) between corrected fLR-PCR and gel electrophoresis (Fig. 3G,H). Allele sizing by fLR-PCR was highly reproducible, as exemplified by an inter-assay variability of less than 0.3% measured on the same set of 4 inter-plate internal controls.

### Validation of the diagnostic approach for detection of *FGF14* GAA repeat expansions (Figs. 1, 2, and Supplementary Fig. S1).

The variability of the methods used thus far to resolve *FGF14* expansions^5, 6^ and the high frequency of GAA-*FGF14* ataxia highlight the need to develop a standardized diagnostic approach that will be accessible and easy to implement in clinical diagnostic laboratories. Such an approach must allow for accurate allele sizing and assessment of repeat motif and sequence interruptions, if any. The former point is particularly important given that (GAA)_250–300_ alleles appear incompletely penetrant^5, 6^. To address this need, we developed and validated a three-step strategy combining (1) fLR-PCR, (2) bidirectional RP-PCRs, and (3) agarose gel electrophoresis of LR-PCR amplification products and Sanger sequencing.

The first step of our proposed strategy involves fLR-PCR amplification of the *FGF14* repeat locus. The identification of two alleles of less than 250 repeat units ends the diagnostic process while any other pattern triggers additional testing. That includes samples with a single normal allele detected on fLR-PCR, since they may carry an expansion larger than 400 repeat units that falls beyond the limit of detection of capillary electrophoresis.

The second step involves bidirectional RP-PCRs targeting the GAA repeat unit at the 5′ end and the 3′ end of the locus. Bidirectional RP-PCRs allow for a comprehensive assessment of the repeat motif over the entire length of expansions and of sequence interruptions and polymorphisms at both ends of the locus. The identification of characteristic saw-toothed products, indicative of a GAA repeat expansion, in a sample found to have at least one allele of 250 or more repeat units on fLR-PCR confirms the diagnosis of GAA-*FGF14* ataxia and ends the diagnostic process. All other samples are moved to the third step.

As part of the third step, gel electrophoresis of LR-PCR products and Sanger sequencing are used to further resolve the remaining samples. First, gel electrophoresis of LR-PCR products is performed on a 1.5% agarose gel for samples with a single allele detected by fLR-PCR, regardless of their RP-PCR profiles. This step allows for distinguishing cases homozygous for two normal alleles from cases heterozygous for one normal allele and one expanded allele that is otherwise too large to be detected by capillary electrophoresis. Gel electrophoresis additionally allows the sizing of large expansions. The motif of any expansion is determined by the RP-PCR profiles generated during step 2, with sawtooth profiles indicating GAA expansions and flat profiles indicating non-GAA expansions. In the case of non-GAA expansions, Sanger sequencing can be performed to determine the repeat motif of the expansion. Second, Sanger sequencing is performed on samples with at least one allele of 250 or more repeat units measured by capillary electrophoresis and a flat or atypical profile on RP-PCRs—indicative of a non-GAA or interrupted expansion—to determine the repeat motif of the *FGF14* locus. Although Sanger sequencing of each strand fails to completely sequence the expanded alleles, thus making this technique unreliable for sizing expanded alleles, their combination allows for sequence coverage of alleles of up to about 500 triplet repeat units.

**Figure 4:**
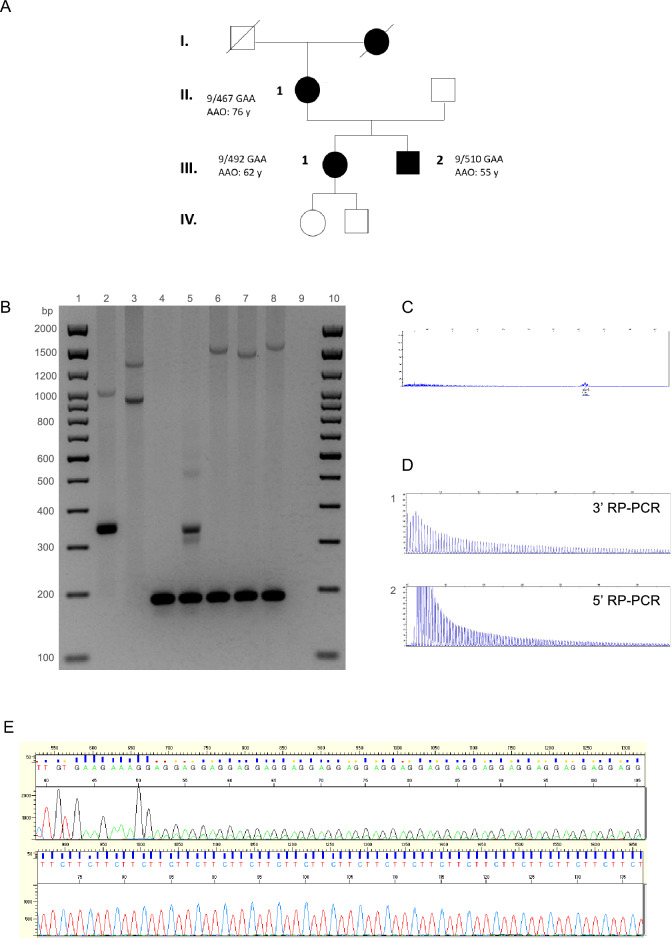
Pedigree and molecular analysis of the *FGF14* repeat locus in a family with GAA-*FGF14* ataxia and a patient compound heterozygous for two expansions. (**A**) Pedigree of an affected multigenerational French family showing autosomal dominant transmission of the disease. Patient II.1 carries a (GAA)_467_ allele and had a disease onset at age 76 years. Patients III.1 and III.2 carry a (GAA)_492_ allele and a (GAA)_510_ allele, respectively. They first manifested episodic ataxia at age 62 years and age 55 years, respectively. AAO indicates age at onset. (**B**) Agarose gel (1.5%), lanes 1 and 10: 2,000 bp molecular weight marker, 2: 58/300 repeat units, 3: 266/417 repeat units, 4: 9/9 repeat units, 5: 9/50 repeat units, 6: 9/492 repeat units, 7: 9/467 repeat units, 8: 9/510 repeat units, and 9: negative control. The agarose gel was imaged with the Uvidoc HD6 Gel Documentation System using the default UV-Gel settings in the Uvitec-1D software, which resulted in overexposure. The gel was cropped for clarity of presentation. The original gel is presented in Supplementary Fig. S11. (**C**) Fluorescent long-range PCR, (**D**) 5′ RP-PCR and 3′ RP-PCR, and (**E**) Sanger sequencing of a patient compound heterozygous for two expanded alleles (266/417 repeat units). (**C**) On fLR-PCR, the smallest expansion of 266 repeat units is detected whereas the larger expansion of 417 repeat units is not as it falls beyond the limit of detection of capillary electrophoresis. (**E**) Sanger sequencing shows polymorphism at the 5′ end of each expansion. Allele 1: (GAA)(GAAA)(GGA)(GAA)_n_ and allele 2: (GAA)(GAAA)(GAAA)(GAA)_n_.

We validated this diagnostic strategy in a cohort of 53 index patients with unsolved LOCA that were recruited in Nancy, France. Using this strategy, we identified nine patients (17%) who carried (GAA)_≥250_ repeat expansions in *FGF14*. The expansion was also present in two affected relatives of one of the index patients (Fig. 4A). We also identified a patient compound heterozygous for expansions of 266 and 417 repeat units (Fig. 4B: lane 3). RP-PCRs accurately determined the repeat motif (GAA and non-GAA expansions) of all expanded alleles in the cohort, as confirmed by forward and reverse Sanger sequencing.

Our diagnostic approach also allowed for the precise characterization of the sequence of each allele in the compound heterozygous patient (Fig. 4B: lane 3, Fig. 4C–E). While the 3′ RP-PCR showed a typical sawtooth pattern, the 5′ RP-PCR showed the superposition of two sawtooth profiles shifted by one base pair (Fig. 4D). These profiles suggested that both GAA repeats were out of phase, as a result of the presence of an insertion or a deletion at the 5′ end of one allele. Sanger sequencing confirmed this hypothesis by showing the presence of a [(GAA)(GAAA)(GGA)] motif at the 5′ end of one allele and a [(GAA)(GAAA)(GAAA)] motif at the 5′ end of the second allele causing both alleles to be out of phase by one base pair.

### Clinical findings

The main clinical features of the French Canadian and French patients with GAA-*FGF14* ataxia are shown in Table 1. Early episodic features, nystagmus (gaze-evoked horizontal and/or downbeat), and alcohol intolerance were reported with similar frequency in patients from both cohorts. Episodic symptoms were reported in 73% of the French Canadian and French patients. The mean (± SD) age at the onset of episodic symptoms was 54 ± 9 years and 64 ± 9 years in the French Canadian and the French patients, respectively. The mean age at the onset of progressive ataxia was 59 ± 10 and 66 ± 10 years, respectively. Nystagmus was observed in 91% and 73% of patients, respectively. More than half of patients reported that alcohol intake triggered episodes of ataxia or markedly worsened baseline ataxia. At the chronic stage, the phenotype was characterized by a slowly progressive pan-cerebellar syndrome with predominant midline involvement. Gait ataxia was observed in 95% and 100% of French Canadian and French patients, respectively. Appendicular ataxia was observed in 90% of French Canadian and 91% of French patients. Pyramidal and extrapyramidal features were less frequently observed (each feature < 20% in the French Canadian cohort and French cohorts). Brain MRI showed mild to moderate cerebellar atrophy, with more severe involvement of the vermis, in 60% and 44% of patients, respectively. We observed an inverse correlation between the size of the repeat expansion and the age at disease onset (22 patients; Pearson correlation coefficient, − 0.46; R^2^ = 0.21; *p* = 0.031) or the age at onset of permanent ataxia (22 patients, Pearson correlation coefficient, − 0.58; R^2^ = 0.34; *p* = 0.005) in the French Canadian cohort. In comparison, the size of the repeat expansion was not significantly associated with age at disease onset (11 patients; Pearson correlation coefficient, 0.07; *p* = 0.85) or the age at onset of permanent ataxia (8 patients, Pearson correlation coefficient, 0.16; *p* = 0.71) in the French cohort.

**Table 1:** Characteristics of the French Canadian and French cohorts.

	French Canadian cohort (*n* = 22)	French cohort (*n* = 11)
*Male sex, no. (%)*	13 (59%)	4 (36%)
*Repeat count of GAA expansion, median (IQR)*	333 (287–392)	370 (313–492)
*Age at onset of episodic symptoms (mean* ± *SD)*	54 ± 9	64 ± 9
*Age at onset of permanent ataxia (mean* ± *SD)*	59 ± 10	66 ± 10
*Inheritance*		
*Familial, no. (%)*	17 (77%)	7 (64%)
*Sporadic, no. (%)*	5 (23%)	4 (36%)
*Episodic onset, no. (%)*	16 (73%)	8 (73%)
*Alcohol intolerance, no. (%)*	9/17 (53%)	5/8 (63%)
*Nystagmus (gaze-evoked horizontal and/or downbeat), no. (%)*	20 (91%)	8 (73%)
*Episodic diplopia or visual blurring, no. (%)*	15/21 (71%)	5/9 (56%)
*Gait ataxia, no. (%)*	21 (95%)	11 (100%)
*Appendicular ataxia, no. (%)*	19/21 (90%)	10 (91%)
*Cerebellar dysarthria, no. (%)*	8/21 (38%)	7 (64%)
*Vertigo or dizziness, no. (%)*	8/20 (40%)	5/10 (50%)
*Cerebellar atrophy on MRI, no. (%)*	12/20 (60%)	4/9 (44%)

No.: number; SD: standard deviation; IQR: interquartile range; MRI: magnetic resonance imaging.

We studied the meiotic stability of the GAA repeat expansion in a French family (Fig. 4A). The transmission of an expanded (GAA)_467_ allele resulted in expansion in the female germline in two meiotic events (expansion from 467 triplets to 492 and 510 triplets) (Fig. 4B: lanes 6, 7, and 8). These results are consistent with previous reports showing further expansion of expanded alleles in the female germline^5, 6^.

Patients III.1 and III.2 developed episodic ataxia and marked alcohol intolerance more than 10 years earlier than their mother (Fig. 4A). Patient III.1 presented with episodic ataxia at age 62 years and developed progressive cerebellar ataxia at age 64 years (SARA score: 4.5 at age 64 years) while patient III.2 presented with episodic ataxia at age 55 years (SARA score: 2 at age 60 years). In comparison, patient II.2 presented with slowly progressive ataxia at age 76 years (SARA score: 23 at age 85 years).

## Discussion

The recent discovery of dominantly inherited GAA repeat expansions in *FGF14*^5, 6^ as a common cause of LOCA highlights the need for developing an accessible and standardized diagnostic protocol. To address this need, we validated a strategy to genotype the *FGF14* GAA repeat expansion that can easily be implemented in clinical settings. This strategy does not rely on long-read sequencing, a technology that is currently not routinely available in clinical laboratories. We used this approach in a cohort of 53 French patients and confirmed the genetic diagnosis in nine patients and two of their relatives.

This step-wise approach was designed to allow for high-throughput screening while not losing sensitivity to detect expansions. This is a particularly desirable point given the large number of patients with unsolved LOCA who are likely to undergo testing for the *FGF14* GAA repeat expansion. The screening process will end after the second step for the majority of samples, as can be seen in Fig. 1. There are, however, a limited number of samples that will need to undergo additional testing, namely gel electrophoresis and Sanger sequencing. Despite being less automatable and more labor-intensive, these tests are necessary to ensure accurate genotyping given the high degree of length and sequence polymorphism of the *FGF14* repeat locus^5^. Although local policies can vary with regard to reporting, suggested items to be included in the report are presented in Supplementary Table S6, depending on the reason for referral.

We also recommend using bidirectional RP-PCRs targeting both ends of the repeat locus during the second step to interrogate the sequence motif over the entire length of larger expansions, which cannot otherwise be achieved with a single RP-PCR. This further allows for a comprehensive assessment of any potential sequence interruptions and polymorphisms at both ends of the locus. While sequence interruptions are relatively common in Friedreich ataxia, an autosomal recessive disorder caused by a GAA repeat expansion in the *FXN* gene^14^, their frequency and impact on disease expression remain to be established in GAA-*FGF14* ataxia. Current data support a pathogenic threshold for GAA-*FGF14* ataxia of (GAA)_≥250_ uninterrupted repeat units^5, 6^. Pending additional data on the effect of interruptions on the pathogenicity of *FGF14* expansions, we suggest that alleles with a minimum repeat tract of 250 uninterrupted triplets be considered pathogenic.

Applying this strategy to a cohort of 53 French patients with unsolved LOCA, we identified nine patients (17%) who carried at least one (GAA)_≥250_ expansion as well as two of their affected relatives. The frequency of the *FGF14* GAA expansion in our cohort is similar to that of other reported cohorts of European descent^5, 6^. Patients from the French Canadian and French cohorts had a similar phenotype that included frequent nystagmus and episodic symptoms at onset followed by a slowly evolving pan-cerebellar syndrome at an average age of 59 years in the French Canadian cohort and 66 years in the French cohort. A substantial proportion of patients also reported marked alcohol intolerance that could precede the development of ataxia by a number of years in some patients. GAA-*FGF14* ataxia is phenotypically similar to SCA6^15^ and the first late-onset episodic ataxia described thus far. Other genetically defined episodic ataxias, such as episodic ataxia type 1 or 2, often manifest in childhood^16–18^. The observation of a cerebellar syndrome of late-onset associated with episodic symptoms, downbeat nystagmus, and alcohol intolerance should prompt testing for the *FGF14* GAA expansion. As with other repeat expansion disorders^19^, we found an inverse correlation between the size of the expansion and the age at onset in the French Canadian cohort. However, we did not observe such a correlation in the French cohort, likely as a result of the small cohort size.

We also found expansion in the female germline across two meiotic events in an affected mother carrying a (GAA)_467_ expansion. These results further highlight the instability of the *FGF14* repeat locus upon meiotic transmission.

The results of this study must be interpreted in light of some limitations. While we tested and validated this diagnostic strategy in two independent patient cohorts, the sample size of both cohorts was limited. The study of larger cohorts in which the performance of this strategy will be orthogonally confirmed by long-read sequencing will be necessary to validate the results presented herein. Further validation of this strategy in other independent diagnostic laboratories will be required as it remains to be established whether the use of other genetic analyzers or capillary electrophoresis instruments may influence the performance of this strategy. We cannot exclude that the modification of any of the analytical parameters used in this study will not influence the performance or results of the molecular strategy. Furthermore, our results cannot exclude the possibility that degradation of DNA from patients carrying *FGF14* expansions may result in allele dropout and false negative results on LR-PCR and RP-PCRs. Sequence polymorphisms at the primer binding sites may similarly result in allele dropout on LR-PCR and RP-PCRs. While we have shown that this strategy can be successfully implemented in clinical diagnostic laboratories, the number of different molecular techniques involved, some of which are time-consuming, may place an additional burden on the technical team of diagnostic laboratories. This is especially relevant given the large shear of patients that are likely to undergo testing for GAA-*FGF14* ataxia.

In conclusion, the recent description of GAA-*FGF14* ataxia as a common cause of LOCA highlighted the need to develop a reliable diagnostic test for this novel condition. To address this need, we developed and validated an approach to diagnosing GAA-*FGF14* ataxia that is reproducible, easy to implement in clinical settings, and does not rely on long-read sequencing. We implemented this protocol in a clinical laboratory and showed its successful application in a cohort of patients with unsolved LOCA.

## Supplementary Information

Supplementary Information 1.

Supplementary Information 2.

## Data Availability

All data generated or analyzed during this study are included in this article and its supplementary information files. The accession number on ClinVar for the *FGF14* variant reported in this paper is SCV003804286. Control DNAs can be shared at the request of any qualified investigator upon reasonable request.
