# Supplementary material for: Optimized testing strategy for the diagnosis of GAA-FGF14 ataxia/spinocerebellar ataxia 27B
Source: Sci Rep. 2023 Jun 15;13:9737. doi: 10.1038/s41598-023-36654-8 (PMC10272173; doi:10.1038/s41598-023-36654-8)
Supplement: Supplementary file 1 — Supplementary Information 1. [file 41598_2023_36654_MOESM1_ESM.pdf]

Supplementary Appendix To:

Optimized testing strategy for the diagnosis of GAA-*FGF14* ataxia/spinocerebellar ataxia 27B

Authors

Céline Bonnet[1,2,†]; David Pellerin[3,4,†]; Virginie Roth[1]; Guillemette Clément[2,5]; Marion Wandzel[1]; Laëtitia Lambert[2,6]; Solène Frismand[5]; Marian Douarinou[5], Anais Grosset[5], Ines Bekkour[5], Frédéric Weber[1]; Florent Girardier[1]; Clément Robin[1]; Stéphanie Cacciatore[1]; Myriam Bronner[1]; Carine Pourié[2]; Natacha Dreumont[2]; Salomé Puisieux[5]; Pablo Iruzubieta[3,7,8,9]; Marie-Josée Dicaire[4]; François Evoy[10]; Marie-France Rioux[10], Armand Hocquel[5]; Roberta La Piana[4,11], Matthis Synofzik[12,13], Henry Houlden[3]; Matt C. Danzi[14]; Stephan Zuchner[14]; Bernard Brais[4,15]; Mathilde Renaud[2,5,6]

†Contributed equally to this work

Affiliations

[1] Laboratoire de Génétique, CHRU de Nancy, Nancy, France

[2] INSERM-U1256 NGERE, Université de Lorraine, Nancy, France

[3] Department of Neuromuscular Diseases, UCL Queen Square Institute of Neurology and The National Hospital for Neurology and Neurosurgery, University College London, London, United Kingdom

[4] Department of Neurology and Neurosurgery, Montreal Neurological Hospital and Institute, McGill University, Montreal, QC, Canada

[5] Service de Neurologie, CHRU de Nancy, Nancy, France

[6] Service de Génétique Clinique, CHRU de Nancy, Nancy, France

[7] Department of Neurology, Donostia University Hospital, San Sebastian, Spain

[8] Neuroscience Area, Biodonostia Health Research Institute, San Sebastian, Spain

[9] Network Center for Biomedical Research in Neurodegenerative Diseases (CIBERNED), Spain

[10] Faculty of Medicine and Health Sciences, Université de Sherbrooke, Sherbrooke, QC, Canada

[11] Department of Diagnostic Radiology, McGill University, Montreal, QC, Canada

[12] Department of Neurodegenerative Diseases, Hertie-Institute for Clinical Brain Research and Center of Neurology, University of Tübingen, Tübingen, Germany

[13] German Center for Neurodegenerative Diseases (DZNE), Tübingen, Germany

[14] Dr. John T. Macdonald Foundation Department of Human Genetics and John P. Hussman Institute for Human Genomics, University of Miami Miller School of Medicine, Miami, FL, USA

[15] Department of Human Genetics, McGill University, Montreal, QC, Canada

Corresponding authors

Dr. Céline Bonnet

Laboratoire de génétique médicale, Hôpitaux de Brabois - CHRU de Nancy

Rue du Morvan, 54500 Vandoeuvre-lès-Nancy FRANCE

ce.bonnet@chru-nancy.fr

Dr. Mathilde Renaud

Service de génétique Clinique, Hôpitaux de Brabois - CHRU de Nancy

Rue du Morvan, 54500 Vandoeuvre-lès-Nancy FRANCE

m.renaud2@chru-nancy.fr

SUPPLEMENTARY METHODS

Genomic DNA extraction methods

Genomic DNA was isolated from peripheral blood in local clinical diagnostic laboratories using a variety of extraction kits as per the manufacturer's instructions: Puregene Blood kit (catalog no. 158026, Qiagen), ReliaPrep Blood gDNA Miniprep System (catalog no. A5082, Promega), NucleoSpin Blood L, Midi kit for DNA from blood using vacuum processing (catalog no. 740954.24, Macherey-Nagel), and NucleoSpin Blood, Mini kit for DNA from blood (catalog no.740951.50, Macherey-Nagel). Genomic DNA was also extracted from saliva, formalin-fixed paraffin-embedded (FFPE) cerebellar tissue, and fresh frozen cerebellar tissue. DNA concentrations were measured with the NanoDrop Spectrophotometer.

Genomic DNA was extracted from saliva using the Oragene•DNA collection kit as per the manufacturer's instructions (catalog no. OG-600, DNA Genotek).

Post-mortem cerebellar tissue was obtained from a patient with GAA-*FGF14* ataxia who had signed an informed consent for brain tissue usage in medical research. Following brain removal, the brain tissue was fixed in 10% buffered formalin and further embedded in paraffin as per standard procedures. Ten 5-micron thick slices of FFPE cerebellar hemisphere were cut and incubated in 1mL of Xylene at 45°C for 15min. The tissue was next centrifuged for 10min at 14,000 rpm and the supernatant was discarded. The tissue pellet was incubated in 1mL of Xylene at 45°C for 15min and centrifuged for 10min at 14,000 rpm. After removing the supernatant and adding 1mL of 100% ethanol to the tissue pellet, the sample was vortexed and centrifuged for 10min at 14,000 rpm. This last step was repeated with 90% ethanol and 70% ethanol. The tissue pellet was next washed twice with phosphate-buffered saline for 15min and incubated overnight at 52°C with 500µL of lysis buffer containing proteinase K, 1M Tris-HCl, 0.5M EDTA, 10% SDS, and distilled water. After adding 500µL of phenol, the sample was vigorously shaken for 5min and centrifuged for 5min at 8,000 rpm. The supernatant was transferred to a new tube, mixed with 500µL of phenol: chloroform: isopropanol (25: 24: 1), and centrifuged for 5 min at 8,000 rpm. This step was repeated once. The supernatant was mixed with 1/10 volume of 3M sodium acetate solution. One equivalent volume of 100% isopropanol was added and the sample was incubated at -20°C overnight. The precipitated DNA was centrifuged at 12,000 rpm at 4°C. The supernatant was discarded and the pellet was washed once with 75% ethanol before being air-dried. The DNA was next resuspended in distilled water and incubated for 2h at 37°C. In addition, genomic DNA was extracted from fresh frozen post-mortem cerebellar hemisphere using the MagAttract HMW DNA kit as per the manufacturer's instructions (catalog no. 67563, Qiagen). Frozen tissues were stored at -80°C until DNA extraction.

Optimization of polymerase chain reaction protocols

The performance of the fluorescent long-range polymerase chain reaction (fLR-PCR) and repeat-primed PCR (RP-PCR) protocols was assessed using different formulations of Taq DNA polymerase. The experiments were performed on DNA specimens from persons carrying an *FGF14* $(GAA)_{\geq 250}$ repeat expansion. We studied the performance of the following 5 Taq DNA polymerases: Phusion Flash High-Fidelity PCR Master Mix 2X (catalog no. F548L, Thermo-Fisher), Qiagen Taq DNA Polymerase (catalog no. 201209, Qiagen), Phusion High-Fidelity DNA Polymerase (catalog no. M0530S, New England BioLabs), GoTaq G2 Hot Start Polymerase (catalog no. M7406, Promega), and ThermoPrime Taq DNA Polymerase (catalog no. AB0301B, Thermo-Fisher). The experimental conditions are shown in Supplementary Table S4.

We next studied the performance of the fLR-PCR and RP-PCR protocols (Supplementary Table S1) along a range of input amounts of genomic DNA: 200ng, 150ng, 100ng, 80ng, 60ng, 50ng, 40ng, 30ng, 20ng, 10ng, and 5ng. DNA concentrations were measured with the NanoDrop Spectrophotometer.

DNA shearing

Genomic DNA specimens from 3 patients carrying an *FGF14* $(GAA)_{371}$, $(GAA)_{383}$, and $(GAA)_{508}$ expansion, respectively, were mechanically sheared using g-TUBES (catalog no. 520079, Covaris) into the smallest possible fragments (~6 kb) as per the manufacturer's instructions. Genomic DNA integrity was assessed pre- and post-shearing using the Genomic DNA ScreenTape protocol on the Agilent 4200 TapeStation instrument (Agilent Technologies).

SUPPLEMENTARY RESULTS

Performance of the PCR protocols with different formulations of Taq DNA polymerase

We compared the performance of the fLR-PCR and RP-PCR protocols on specimens from persons carrying an *FGF14* GAA expansion using 5 different formulations of Taq DNA polymerase. Our findings showed that the Phusion Flash High-Fidelity PCR Master Mix and the GoTaq G2 Hot Start Polymerase performed best on fLR-PCR of expanded *FGF14* alleles, as assessed by relative fluorescence units (RFU) peak intensity (Supplementary Figure S2). However, compared to the Phusion Flash High-Fidelity PCR Master Mix, the GoTaq G2 Hot Start Polymerase provided less consistent amplification of expanded alleles. The Phusion High-Fidelity DNA Polymerase and the ThermoPrime Taq DNA Polymerase yielded low to undetectable RFU peak intensity of expanded alleles. Furthermore, we also found that the Phusion Flash High-Fidelity PCR Master Mix performed best on RP-PCRs of expanded alleles compared to other Taq DNA polymerase formulations, which yielded less well-defined and resolute electrophoretic profiles of lower intensity (Supplementary Figure S2). Overall, our results showed that the Phusion Flash High-Fidelity PCR Master Mix was the only Taq DNA polymerase studied that performed well and consistently in both fLR-PCR and RP-PCR protocols.

Effect of different input amounts of genomic DNA on the performance of the PCR protocols

We assessed the performance of the fLR-PCR and RP-PCR protocols along a range of input amounts of genomic DNA, ranging from 200ng to 5ng, using specimens from 3 persons respectively carrying an *FGF14* $(GAA)_{262}$, $(GAA)_{311}$, and $(GAA)_{404}$ repeat expansion. The fLR-PCR protocol yielded consistent amplification of expanded alleles with all DNA input amounts tested (Supplementary Figure S3). The 5' RP-PCR yielded high-intensity saw-toothed profiles with all input amounts of DNA tested, although less consistently with 5ng of input DNA (Supplementary Figure S3). In comparison, we found the 3' RP-PCR to perform best and yield consistent saw-toothed profiles with DNA input amounts of at least 20ng (Supplementary Figure S3). Lower input amounts in 3' RP-PCR yielded low-intensity to undetectable saw-toothed profiles.

Performance of the PCR protocols on genomic DNA extracted from different tissues

The genomic DNA isolated from peripheral blood of patients included in this study was extracted using one of four different extraction kits, depending on the local clinical laboratory performing the extraction as listed in the Supplementary Methods. None of the extraction kits affected the performance of the PCR protocols negatively. We also assessed the performance of the LR-PCR and RP-PCR protocols using DNA extracted from saliva, FFPE

cerebellar cortex, and fresh frozen cerebellar cortex. We used DNA extracted from the saliva of a person with *FGF14* $(GAA)_{11/354}$ alleles, DNA extracted from FFPE cerebellar cortex of a person with *FGF14* $(GAA)_{9/474}$ alleles, and DNA extracted from fresh frozen cerebellar cortex of a person with *FGF14* $(GAA)_{9/331}$ alleles. We found that the expanded alleles of DNA extracted from saliva and fresh frozen cerebellar tissue were adequately resolved by LR-PCR and RP-PCRs, while the expanded allele of DNA extracted from FFPE cerebellar cortex could not be resolved by any PCR technique (Supplementary Figure S4).

Assessment of the effect of DNA shearing on the performance of the PCR protocols

Our objective was to determine whether shearing DNA from patients with an *FGF14* GAA expansion could cause false negative results on LR-PCR and RP-PCRs. We sheared genomic DNA from 3 patients carrying an *FGF14* $(GAA)_{371}$, $(GAA)_{383}$, and $(GAA)_{508}$ expansion, respectively, and assessed DNA integrity using the Agilent 4200 TapeStation instrument (Supplementary Figure S5). We found that shearing did not result in allele dropout and false negative results on LR-PCR, fLR-PCR, and RP-PCR, although RFU peak intensity of sheared samples was lower than that of non-sheared samples on capillary electrophoresis (Supplementary Figure S6). However, these results do not rule out the possibility that DNA degradation in patients with *FGF14* expansions could lead to allele dropout and false negative results on LR-PCR and RP-PCRs.

Comparison of GeneMapper and Peak Scanner software

We compared the performance of GeneMapper (version 6.0, Applied Biosystems) and Peak Scanner (version 3.0, Applied Biosystems) to analyze the results of capillary electrophoresis. Results were analyzed using the built-in microsatellite default settings in GeneMapper and the default settings in Peak Scanner. We found both software to be suitable for the analysis of capillary electrophoresis results of LR-PCR and RP-PCRs (Supplementary Figure S7). In both software, high molecular weight peaks associated with alleles $(GAA)_{\geq 250}$ repeat units had a characteristic bell-shaped appearance that was readily distinguishable from the background on capillary electrophoresis of fluorescent LR-PCR amplification products (see Figure 2, Figure 4, and Supplementary Figure S7). The relative fluorescence units (RFU) peak intensity of these high molecular weight peaks generally varied between 200 and 30 RFU, decrementing with increasing peak size. Expansions $(GAA)_{>400}$ repeat units could not be accurately sized with both software as they fall beyond the limit of detection of capillary electrophoresis. In comparison, the RFU of lower molecular weight peaks generally varied between 30,000 RFU for the shortest alleles carrying 8 GAA repeat units and 200 RFU for alleles carrying 250 GAA repeat units.

We also observed that compared to $(GAA)_n$ repeat expansions, fluorescent LR-PCR amplification products of $(GAAGGA)_n$ and $[(GAA)_4(GCA)_1]_n$ repeat expansions produced a cluster of non-bell-shaped peaks on capillary electrophoresis (Supplementary Figure S8). This observation needs confirmation on a larger number of non-GAA expansions. It also remains to be established if other non-GAA motifs yield similar non-bell-shaped peaks on capillary electrophoresis.

SUPPLEMENTARY FIGURES

Supplementary Figure S1: Testing strategy for the diagnosis of GAA-*FGF14* ataxia

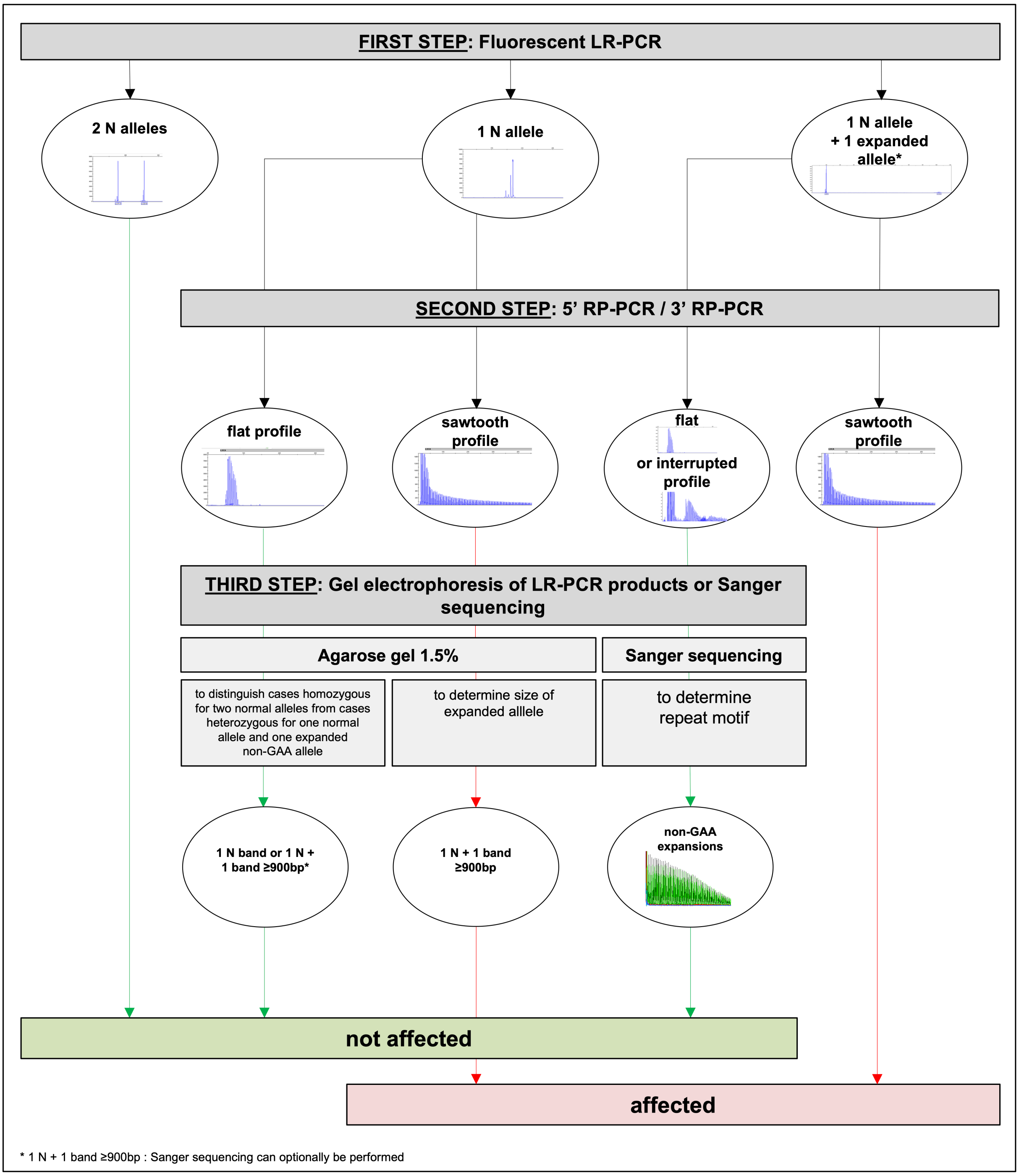

Normal alleles have $(GAA)_{<250}$ repeats and expanded alleles have $(GAA)_{\geq 250}$ repeats.

*Two expanded alleles are possible

Legend: N allele, normal allele <250 repeat units; LR-PCR, long-range polymerase chain reaction; RP-PCR, repeat-primed PCR.

Supplementary Figure S2: Performance of the PCR protocols with different formulations of Taq DNA polymerase

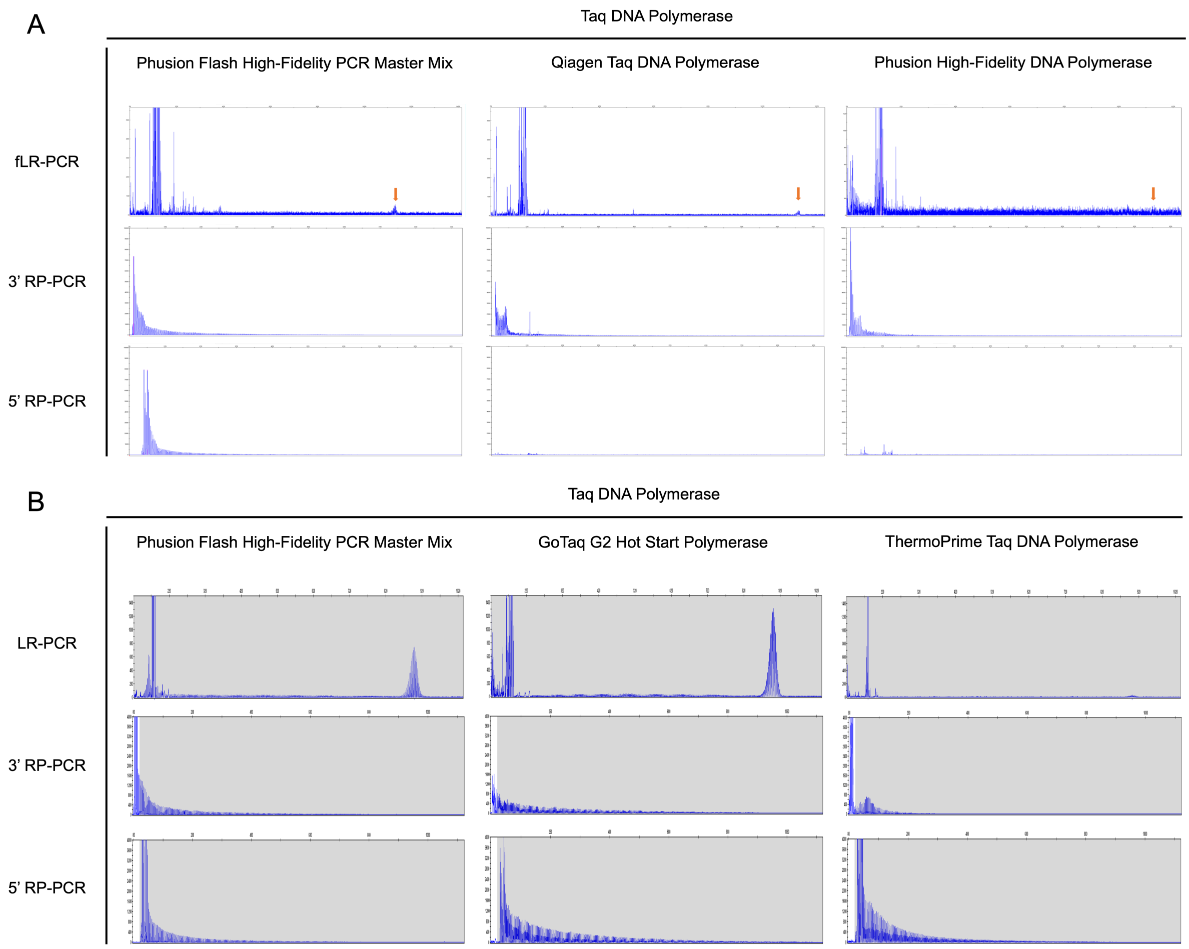

(A) Comparison of the performance of the Phusion Flash High-Fidelity PCR Master Mix, Qiagen Taq DNA Polymerase, and Phusion High-Fidelity DNA Polymerase in fluorescent long-range PCR (fLR-PCR) and repeat-primed PCR (RP-PCR) protocols. Representative LR-PCR and RP-PCR chromatograms obtained by analyzing the specimen from a person carrying an *FGF14* $(GAA)_{383}$ expansion are shown. The orange arrows show high molecular weight peaks associated with the expanded *FGF14* allele detected by capillary electrophoresis. The relative fluorescence units peak intensity of the fLR-PCR traces has been adjusted to optimize visualization of the high molecular weight peaks. All RP-PCR traces are displayed at 10,000 relative fluorescence units.

(B) Comparison of the performance of the Phusion Flash High-Fidelity PCR Master Mix, GoTaq G2 Hot Start Polymerase, and ThermoPrime Taq DNA Polymerase in fLR-PCR and RP-PCR protocols. Representative LR-PCR and RP-PCR chromatograms obtained by analyzing the specimen from a person carrying an *FGF14* $(GAA)_{271}$ expansion are shown. The fLR-PCR and RP-PCR traces are displayed at 1,400 and 4,000 relative fluorescence units, respectively.

Supplementary Figure S3: Performance of the PCR protocols with different input amounts of genomic DNA

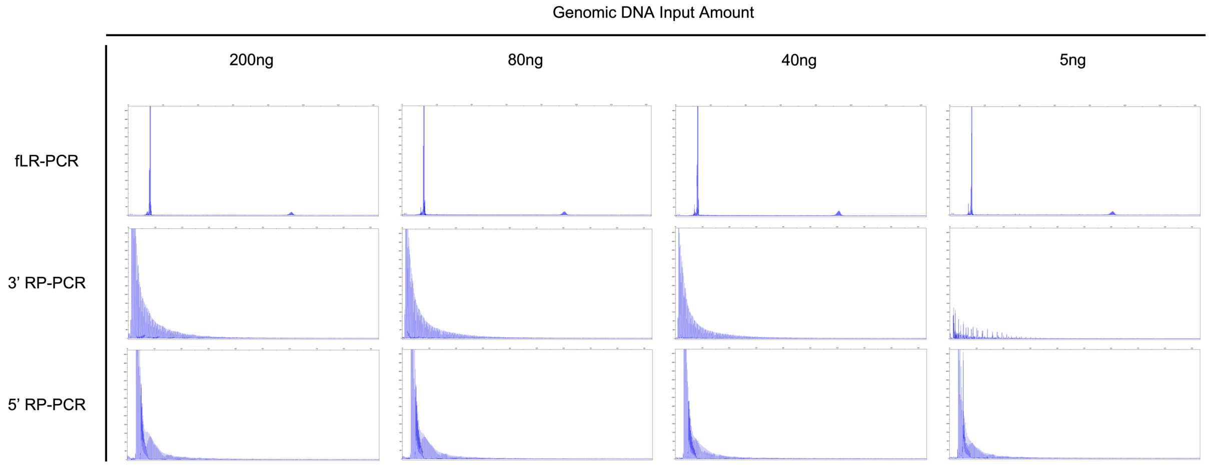

Comparison of the performance of the fluorescent long-range PCR (fLR-PCR) and repeat-primed PCR (RP-PCR) protocols along a range of input amounts of genomic DNA. The results obtained with 200ng, 80ng, 40ng, and 5ng of input amounts of DNA are shown. Representative LR-PCR and RP-PCR chromatograms obtained by analyzing the specimen from a person carrying an *FGF14* $(GAA)_{311}$ expansion are shown. All traces are displayed at 5,000 relative fluorescence units.

Supplementary Figure S4: Performance of the PCR protocols on genomic DNA extracted from different tissues

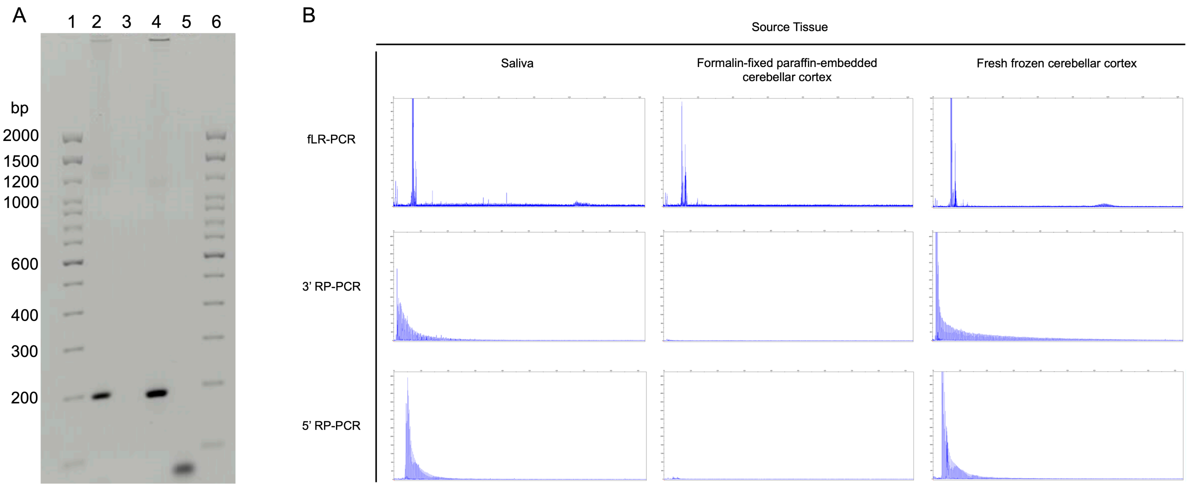

(A) Agarose gel electrophoresis (1.5%), lanes 1 and 6: 2,000 bp molecular weight marker, 2: genomic DNA extracted from saliva, 3: genomic DNA extracted from formalin-fixed paraffin-embedded (FFPE) cerebellar cortex, 4: genomic DNA extracted from fresh frozen cerebellar cortex, and 5: negative template control. The agarose gel was imaged with the Uvidoc HD6 Gel Documentation System using the default UV-Gel settings in the Uvitec-1D software, which resulted in overexposure of the agarose gel.

(B) Comparison of the performance of the fluorescent long-range PCR (fLR-PCR) and repeat-primed PCR (RP-PCR) protocols with input genomic DNA extracted from saliva, FFPE cerebellar cortex, and fresh frozen cerebellar cortex. Representative LR-PCR and RP-PCR chromatograms obtained by analyzing the saliva-extracted DNA specimen from a person with *FGF14* $(GAA)_{11/354}$ alleles, FFPE cerebellar cortex-extracted DNA specimen from a person with *FGF14* $(GAA)_{9/474}$ alleles, and fresh frozen cerebellar cortex-extracted DNA specimen from a person with *FGF14* $(GAA)_{9/331}$ alleles. The relative fluorescence units peak intensity of the fLR-PCR traces has been adjusted to optimize visualization of the high molecular weight peaks, which were not detectable with amplification of DNA extracted from FFPE cerebellar cortex. RP-PCR traces are displayed at 5,000 relative fluorescence units.

Supplementary Figure S5: Integrity of genomic DNA pre- and post-shearing

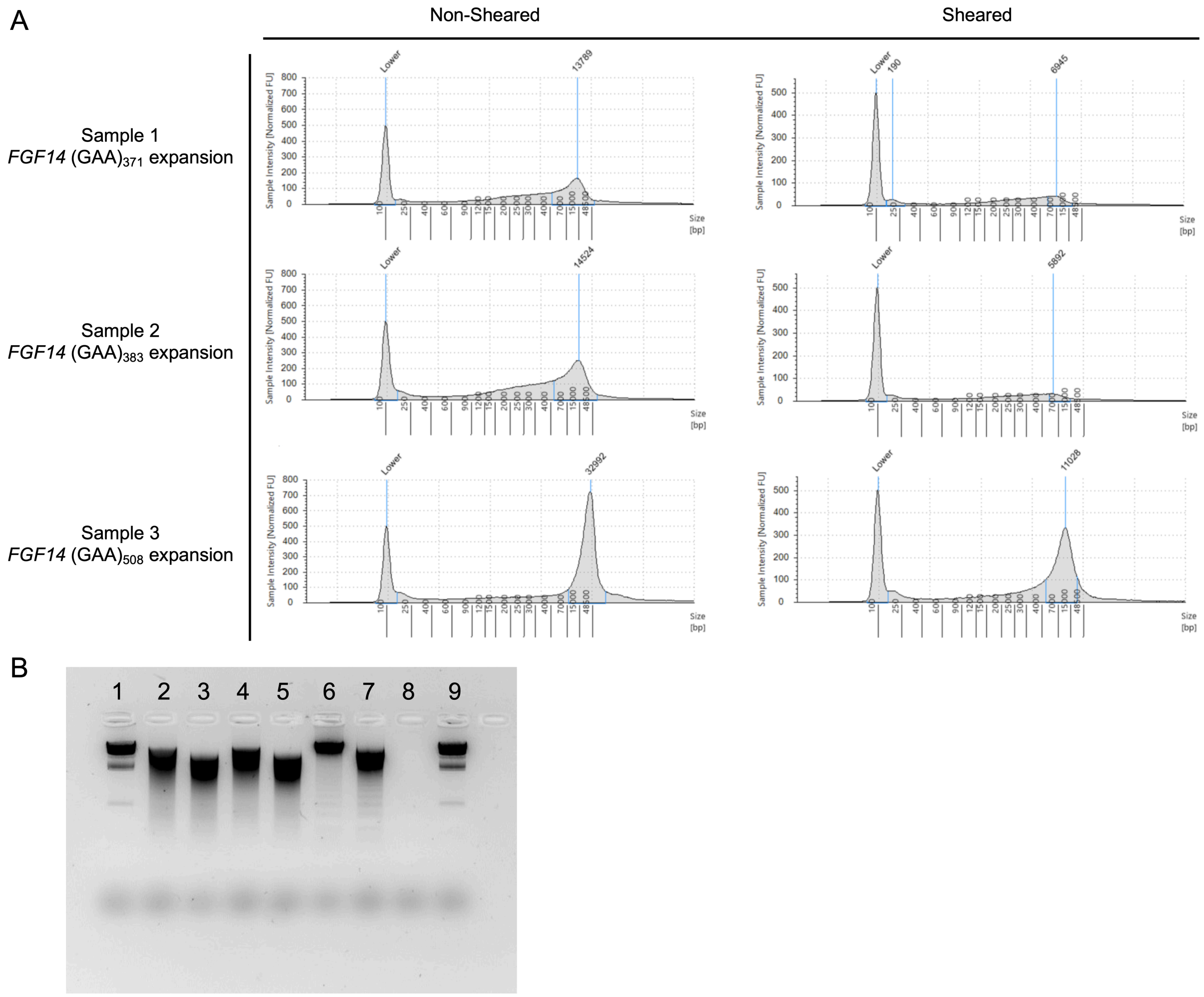

(A) Electropherograms of non-sheared and sheared samples obtained using the Genomic DNA ScreenTape protocol on the Agilent 4200 TapeStation instrument. The mean DNA fragment size was 14kb pre-shearing and 7kb post-shearing for sample 1, 15kb pre-shearing and 6kb post-shearing for sample 2, and 33kb pre-shearing and 11kb post-shearing for sample 3.

(B) Agarose gel electrophoresis (0.8%), lanes 1 and 9: λ DNA/HindIII marker, 2: non-sheared DNA and 3: sheared DNA from sample 1, 4: non-sheared DNA and 5: sheared DNA from sample 2, 6: non-sheared DNA and 7: sheared DNA from sample 3, and 8: negative control. Five microliters of DNA at a concentration of 40ng/uL was loaded on agarose gel. The gel was migrated at 130V for 30 minutes and resolved with the Uvidoc HD6 Gel Documentation System.

Supplementary Figure S6: Effect of DNA shearing on the performance of the PCR protocols

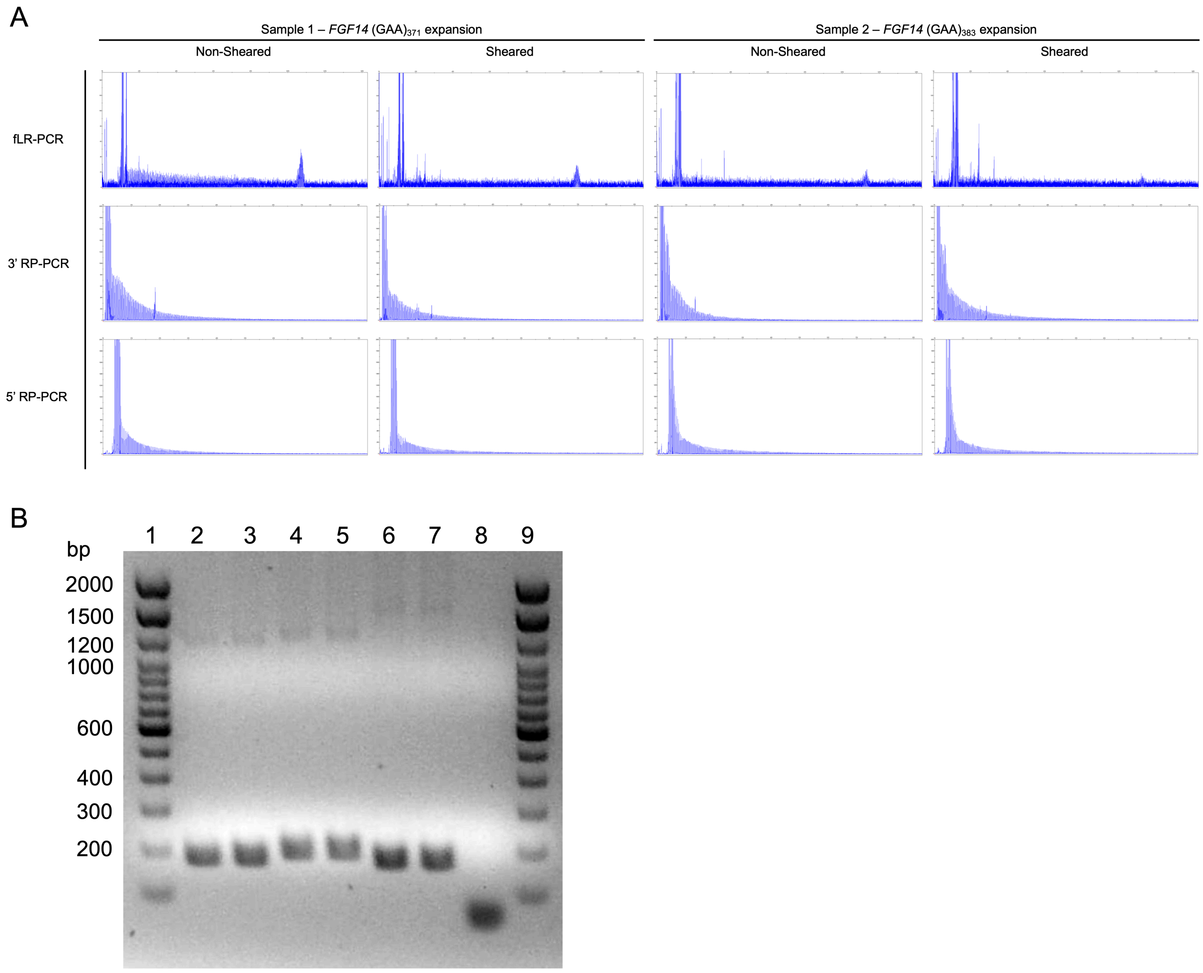

(A) Comparison of the performance of the fluorescent long-range PCR (fLR-PCR) and repeat-primed PCR (RP-PCR) protocols using sheared and non-sheared genomic DNA. Representative LR-PCR and RP-PCR chromatograms obtained by analyzing the genomic DNA from a person carrying an *FGF14* $(GAA)_{371}$ expansion and from a person carrying an *FGF14* $(GAA)_{383}$ expansion. The fLR-PCR traces and RP-PCR traces are displayed at 150 and 2,000 relative fluorescence units, respectively.

(B) Agarose gel electrophoresis (1.5%), lanes 1 and 9: 2,000 bp molecular weight marker, 2: LR-PCR amplification products of non-sheared DNA and 3: sheared DNA from sample 1, 4: LR-PCR amplification products of non-sheared DNA and 5: sheared DNA from sample 2, 6: LR-PCR amplification products of non-sheared DNA and 7: sheared DNA from sample 3, and 8: negative template control. The agarose gel was migrated at 150V for 35 minutes and resolved with the AlphaDigidoc Imaging System using default settings.

Supplementary Figure S7: Comparison of GeneMapper and Peak Scanner software

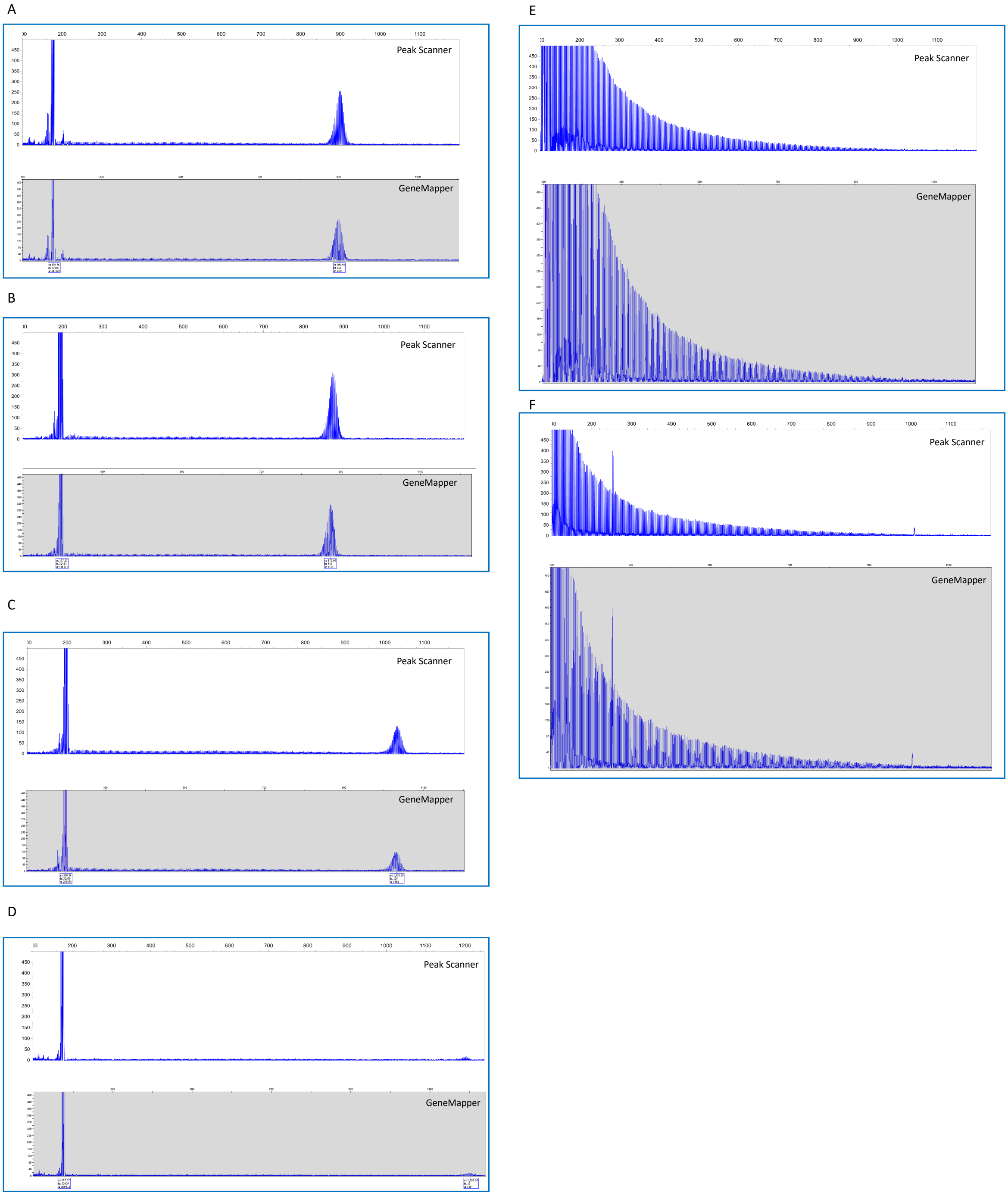

Analysis of capillary electrophoresis results by Peak Scanner (v3.0) and GeneMapper (v6.0). (A-D) Representative fLR-PCR traces of patients carrying an *FGF14* GAA expansion analyzed by Peak Scanner and GeneMapper. Representative (E) 5' RP-PCR and (F) 3' RP-PCR traces of a patient carrying an *FGF14* GAA expansion analyzed by Peak Scanner and GeneMapper.

Supplementary Figure S8: Long-range PCR electrophoretic profiles of GAA and non-GAA expansions

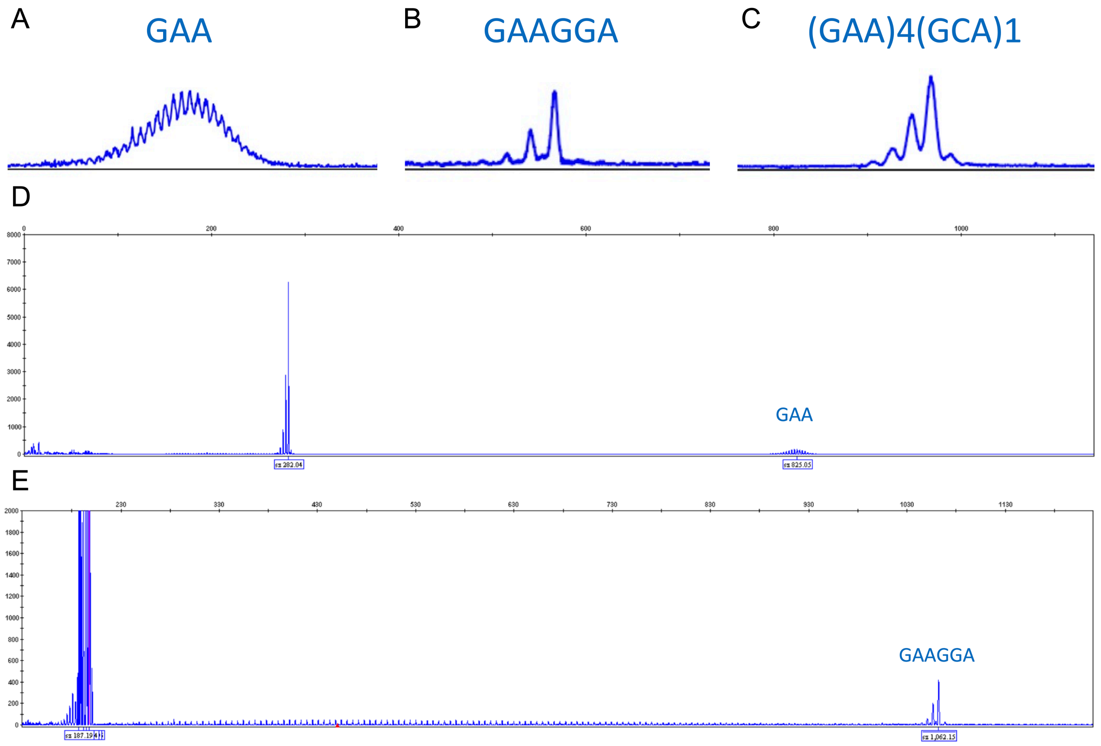

(A-C) Fluorescent long-range PCR electrophoretic profiles of GAA expansions, GAAGGA expansions, and [$(GAA)_4(GCA)_1$] expansions. (D) Representative fLR-PCR trace showing the characteristic bell-shaped appearance of a GAA repeat expansion, and (E) representative fLR-PCR trace showing a cluster of non-bell-shaped peaks associated with a GAAGGA repeat expansion.

Supplementary Figure S9: *FGF14* allele size estimates by long-read nanopore sequencing and agarose gel electrophoresis.

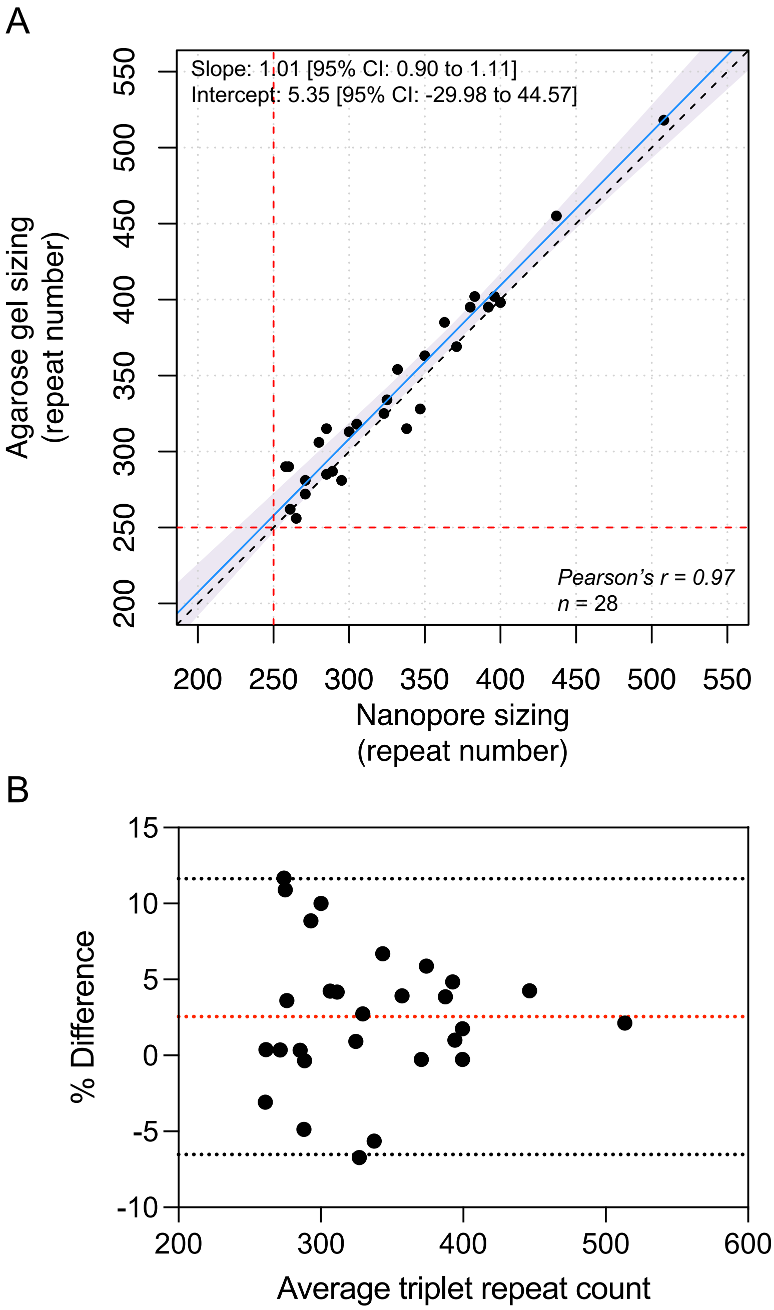

(A) Passing-Bablok regression (blue line) with 95% confidence interval (shaded blue area) for allele size measured by nanopore sequencing and gel electrophoresis. The dashed black line shows the identity line and the dashed red lines show the pathogenic threshold of $(GAA)_{\geq 250}$ repeats. (B) Bland-Altman plot shows the percentage difference between size estimates measured by targeted nanopore sequencing and gel electrophoresis as a function of the average of the two measurements for each sample. The dashed red line shows the mean bias between the two techniques and the dashed gray lines show the limits of agreement, defined as the mean percentage difference ± 1.96SD.

Supplementary Figure S10: Uncropped original agarose gels to Figure 2D-E

A B

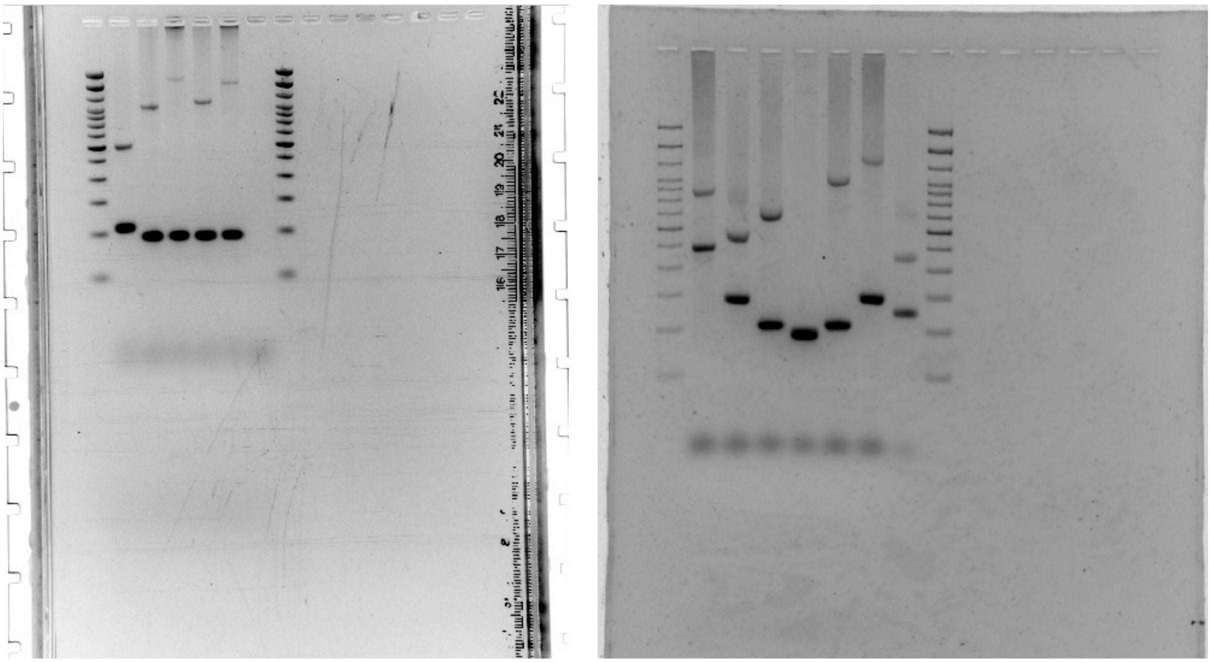

(A) Original image to Figure 2D. Agarose gel electrophoresis (1.5%), lanes 1 and 8: 2,000 bp molecular weight marker, 2: 17/132 repeat units, 3: 9/284 repeat units, 4: 9/510 repeat units, 5: 9/313 repeat units, 6: 9/467 repeat units; 7: negative control.

(B) Original image to Figure 2E. Agarose gel electrophoresis (1.5%), lanes 1 and 9: 2,000 bp molecular weight marker, 2: 107/236 repeat units, 3: 40/132 repeat units, 4: 16/182 repeat units, 5: 8/9 repeat units, 6: 17/319 repeat units; 7: 43/370 repeat units, 8: 28/94 repeat units.

The agarose gels were imaged with the Uvidoc HD6 Gel Documentation System using the default UV-Gel settings in the Uvitec-1D software, which resulted in overexposure.

Supplementary Figure S11: Uncropped original agarose gel to Figure 4B

A

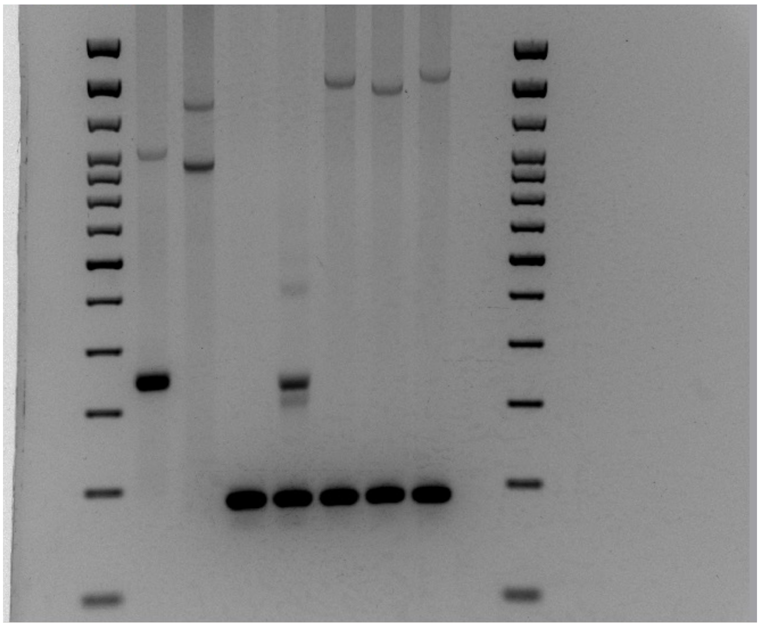

(A) Original image to Figure 4B. Agarose gel (1.5%), lanes 1 and 10: 2,000 bp molecular weight marker, 2: 58/300 repeat units, 3: 266/417 repeat units, 4: 9/9 repeat units, 5: 9/50 repeat units, 6: 9/492 repeat units, 7: 9/467 repeat units, 8: 9/510 repeat units, 9: negative control. The agarose gel was imaged with the Uvidoc HD6 Gel Documentation System using the default UV-Gel settings in the Uvitec-1D software, which resulted in overexposure.

SUPPLEMENTARY TABLES

Supplementary Table S1: Experimental conditions used for long-range PCR and RP-PCR protocols

Long-range PCR protocols

Reagents	Primers	Cycling conditions
1. Phusion Flash High-Fidelity PCR Master Mix 2X (Thermo-Fisher)<br>2. Primers 1µM<br>3. gDNA 40ng	**LR-PCR**<br>F: TGCAAATGAAGGAAAACTCTT<br>R: CAATGATGAATTAAGCAGTTCC<br><br>**fLR-PCR**<br>F: 6-FAM-TGCAAATGAAGGAAAACTCTT<br>R: CAATGATGAATTAAGCAGTTCC	98°C x 3 min<br>[98°C x 10 sec<br>65°C x 15 sec – Each 2 cycles decreasing by 1°C<br>72°C x 3 min] x 12 cycles<br><br>[98°C x 10 sec<br>59°C x 15 sec<br>72°C x 3 min] x 20 cycles<br><br>72°C x 5 min

RP-PCR protocols

Reagents	Primers	Cycling conditions
1. Phusion Flash High-Fidelity PCR Master Mix 2X (Thermo-Fisher)<br>2. F primer 1µM<br>3. R primer 0.1µM<br>4. M13-FAM 1µM<br>5. gDNA 40ng	**3'RP-PCR**<br>F: TGCCCACATAGAGCTTAGTCT<br>R: CACGACGTTGTAAAACGAC-GAAGAAGAAGAAGAAGAAGAA<br>M13-FAM: 6-FAM-CACGACGTTGTAAAACGAC<br><br>**5'RP-PCR**<br>F: TGCAAATGAAGGAAAACTCTT<br>R: CACGACGTTGTAAAACGAC-TTCTTCTTCTTCTTCTTCTTC<br>M13-FAM: 6-FAM-CACGACGTTGTAAAACGAC	98°C x 3 min<br><br>[98°C x 10 sec<br>65°C x 15 sec<br>72°C x 1 min] x 35 cycles<br><br>72°C x 5 min

Fragment length analysis (for long-range PCR and RP-PCRs)

Reagents	Volume
GeneScan 1200 Liz Dye Size Standard (Applied Biosystems)	0.5 µL
HiDi-Formamide (Applied Biosystems)	9 µL
PCR amplification products	0.5 µL

F, forward; fLR-PCR, fluorescent long-range polymerase chain reaction; gDNA, genomic DNA; LR-PCR, long-range polymerase chain reaction; RP-PCR, repeat-primed polymerase chain reaction; R, reverse.

Supplementary Table S2: Run module parameters used for capillary electrophoresis

ABI 3130xl and ABI 3730xl DNA Analyzers

Parameter	Value
Oven Temperature	63°C
Buffer Temperature	35°C
Pre-run Voltage	15 kV
Pre-run Time	180 sec
Injection Voltage	1.6 kV
Injection Time	5sec/15sec/30sec
First Readout Time	200 msec
Second Redout Time	200 msec
Run Voltage	8 kV
Voltage Number of Steps	10 steps
Voltage Step Interval	20 secs
Temperature Step	0.2 secs
Voltage Tolerance	0.6 kV
Current Stability	30 uA
Ramp Delay	1 sec
Data Delay	350 sec
Run Time	7000 sec

ABI 3500xl DNA Analyzers

Parameter	Value
Oven Temperature	63°C
Pre-run Voltage	15 kV
Pre-run Time	180 sec
Injection Voltage	1.6 kV
Injection Time	5sec/15sec/30sec
First Readout Time	180 msec
Second Redout Time	180 msec
Run Voltage	8 kV
Voltage Number of Steps	40 steps
Voltage Step Interval	15 secs
Voltage Tolerance	0.6 kV
Current Stability	30 uA
Data Delay	480 sec
Run Time	7000 sec

Supplementary Table S3: Sanger sequencing protocol

Purification protocol

Reagents	Volume	Cycling conditions
1. Illustra ExoProStar, (catalog no. GEUS78210, Cytiva)<br>2. PCR amplification products diluted 1:4	1 µL<br><br>5 µL	37°C x 15 min<br><br>80°C x 15 min<br><br>4°C hold

Sequencing – Step 1

Reagents	Primers	Cycling conditions
1. BigDye Terminator 2µL (Applied Biosystems)<br>2. Sequencing Buffer 5X 1µL<br>3. Primer (F or R) 5µM 1µL<br>4. Purified PCR products 1µL<br>5. H_2O 5µL	F: TGCAAATGAAGGAAAACTCTT<br>R: CAATGATGAATTAAGCAGTTCC	96°C x 1 min<br><br>[96°C x 10 sec<br>55°C x 5 sec<br>60°C x 4 min] x 25 cycles<br><br>4°C hold

Sequencing – Step 2

Reagents	Protocol
1. BigDye XTerminator 10µL (Applied Biosystems)<br>2. SAM Solution 45µL (Applied Biosystems)<br>3. Sequencing products diluted 1:2 5µL<br>4. H_2O 5µL	1. Vortex x 30 min (room temperature)<br>2. Centrifuge 1,000*g* x 2 min

Sequencing – Step 3

Applied Biosystems 3130*xl* DNA Analyzer

1. Injection time: 6 seconds
2. Run time: 6,000 seconds

Supplementary Table S4: Experimental conditions used with different formulations of Taq DNA polymerase

Long-range PCR protocols

**Taq DNA polymerase formulations**	**Primers**	**Cycling conditions**
Phusion Flash High-Fidelity PCR Master Mix 2X<br>1. Phusion Flash High-Fidelity PCR Master Mix 2X (Thermo-Fisher)<br>2. Primers 1μM<br>3. gDNA 40ng<br><br>*Qiagen Taq DNA Polymerase*<br>1. Taq Polymerase 0.75U<br>2. Q-Solution 5X<br>3. PCR Buffer 10X<br>4. dNTP 0.125mM<br>5. Primers 1μM<br>6. gDNA 80ng<br><br>*Phusion High-Fidelity DNA Polymerase*<br>1. Phusion High-Fidelity DNA Polymerase 0.4U<br>2. Phusion HF Buffer 5X<br>3. dNTP 0.2mM<br>4. Primers 0.5μM<br>5. gDNA 80ng<br><br>*GoTaq G2 Hot Start Polymerase*<br>1. GoTaq G2 Hot Start Polymerase 1U<br>2. GoTaq Flexi Buffer 5X<br>3. $MgCl_2$ 1.5mM<br>4. dNTP 0.2mM<br>5. Primers 1μM<br>6. gDNA 40ng<br><br>*ThermoPrime Taq DNA Polymerase*<br>1. ThermoPrime Taq DNA Polymerase 1U<br>2. Reaction Buffer IV 10X<br>3. $MgCl_2$ 1.5mM<br>4. dNTP 0.2mM<br>5. Primers 1μM<br>6. gDNA 40ng	**LR-PCR**<br>F: TGCAAATGAAGGAAAACTCTT<br>R: CAATGATGAATTAAGCAGTTCC<br><br>**fLR-PCR**<br>F: 6-FAM-TGCAAATGAAGGAAAACTCTT<br>R: CAATGATGAATTAAGCAGTTCC	98°C x 3 min<br>[98°C x 10 sec<br>65°C x 15 sec – Each 2 cycles decreasing by 1°C<br>72°C x 3 min] x 12 cycles<br><br>[98°C x 10 sec<br>59°C x 15 sec<br>72°C x 3 min] x 20 cycles<br><br>72°C x 5 min

RP-PCR protocols

**Taq DNA polymerase formulations**	**Primers**	**Cycling conditions**
Phusion Flash High-Fidelity PCR Master Mix 2X<br>1. Phusion Flash High-Fidelity PCR Master Mix 2X (Thermo-Fisher)<br>2. F primer 1μM<br>3. R primer 0.1μM<br>4. M13-FAM 1μM<br>5. gDNA 40ng	**3'RP-PCR**<br>F: TGCCCACATAGAGCTTAGTCT<br>R: CACGACGTTGTAAAACGAC-GAAGAAGAAGAAGAAGAAGAA<br>M13-FAM: 6-FAM-CACGACGTTGTAAAACGAC<br><br>**5'RP-PCR**<br>F: TGCAAATGAAGGAAAACTCTT<br>R: CACGACGTTGTAAAACGAC-TTCTTCTTCTTCTTCTTCTTC<br>M13-FAM: 6-FAM-CACGACGTTGTAAAACGAC	98°C x 3 min<br><br>[98°C x 10 sec<br>65°C x 15 sec<br>72°C x 1 min] x 35 cycles<br><br>72°C x 5 min

Qiagen Taq DNA Polymerase<br>1. Taq Polymerase 0.75U<br>2. Q-Solution 5X<br>3. PCR Buffer 10X<br>4. dNTP 0.125mM<br>5. F primer 1μM<br>6. R primer 0.1μM<br>7. M13-FAM 1μM<br>8. gDNA 80ng<br><br>*Phusion High-Fidelity DNA Polymerase*<br>1. Phusion High-Fidelity DNA Polymerase 0.4U<br>2. Phusion HF Buffer 5X<br>3. dNTP 0.2mM<br>4. F primer 0.5μM<br>5. R primer 0.05μM<br>6. M13-FAM 0.5μM<br>7. gDNA 80ng<br><br>*GoTaq G2 Hot Start Polymerase*<br>1. GoTaq G2 Hot Start Polymerase 1U<br>2. GoTaq Flexi Buffer 5X<br>3. $MgCl_2$ 1.5mM<br>4. dNTP 0.2mM<br>5. F primer 1μM<br>6. R primer 0.1μM<br>7. M13-FAM 1μM<br>8. gDNA 40ng (3' RP-PCR) or 80ng (5' RP-PCR)<br><br>*ThermoPrime Taq DNA Polymerase*<br>1. ThermoPrime Taq DNA Polymerase 1U<br>2. Reaction Buffer IV 10X<br>3. $MgCl_2$ 1.5mM<br>4. dNTP 0.2mM<br>5. F primer 1μM<br>6. R primer 0.1μM<br>7. M13-FAM 1μM<br>8. gDNA 40ng (3' RP-PCR) or 80ng (5' RP-PCR)		

F, forward; fLR-PCR, fluorescent long-range polymerase chain reaction; gDNA, genomic DNA; LR-PCR, long-range polymerase chain reaction; RP-PCR, repeat-primed polymerase chain reaction; R, reverse.

Supplementary Table S5: Comparison of sizing estimates by fluorescent LR-PCR (with and without correction) and targeted nanopore sequencing

Nanopore sizing	fluorescent LR-PCR sizing				
repeat number [size bp]	[size bp]	repeat number calculated		corrected	
258	876.90	242.30	-6%	266.02	3%
260	873.71	241.24	-7%	264.83	2%
261	879.92	243.31	-7%	267.14	2%
265	874.36	241.45	-9%	265.07	0%
271	900.00	250.00	-8%	274.63	1%
271	905.57	251.86	-7%	276.70	2%
280	921.94	257.31	-8%	282.81	1%
285	955.35	268.45	-6%	295.26	4%
285	929.72	259.91	-9%	285.71	0%
289	952.90	267.63	-7%	294.35	2%
295	936.54	262.18	-11%	288.25	-2%
300	1035.55	295.18	-2%	325.15	8%
305	1003.79	284.60	-7%	313.31	3%
323	1039.49	296.50	-8%	326.62	1%
325	1057.28	302.43	-7%	333.25	3%
332	1060.24	303.41	-9%	334.36	1%
338	1041.80	297.27	-12%	327.48	-3%
347	1062.12	304.04	-12%	335.06	-3%
350	1121.00	323.67	-8%	357.01	2%
363	1173.86	341.29	-6%	376.71	4%
371	1153.54	334.51	-10%	369.13	-1%
380	1215.20	355.07	-7%	392.12	3%
383	1208.65	352.88	-8%	389.68	2%
392	1228.55	359.52	-8%	397.09	1%
396	1220.51	356.84	-10%	394.10	0%
400	1214.87	354.96	-11%	392.00	-2%

Slope: 2.6827; y-intercept: 163.2416
Internal control: $(GAA)_{10}$: 180,32 bp; $(GAA)_{258}$:876.90 bp; $(GAA)_{323}$:1039.69 bp; $(GAA)_{400}$:1214.87 bp.

Supplementary Table S6: Suggested items to be included in the report

Range: number of repeats	Reason for referral	
	**Diagnostic testing**	**Carrier testing**
**Normal**<br>-Allele 1 <250 repeat units<br>-Allele 2 <250 repeat units	As per currently established diagnostic thresholds, the diagnosis of GAA-*FGF14* (SCA27B) ataxia is excluded	As per currently established diagnostic thresholds, the patient is not a carrier of a *FGF14* GAA repeat expansion
**Non-GAA expansion: ≥250 repeat units**<br>-Allele 1 <250 repeat units<br>-Allele 2 ≥250 non-GAA repeat units	As per currently established diagnostic thresholds, the diagnosis of GAA-*FGF14* ataxia (SCA27B) is excluded	-
**GAA expansion: ≥250 repeat units**<br>-Allele 1 <250 repeat units<br>-Allele 2 ≥250 GAA repeat units<br>or<br>-Allele 1 ≥250 GAA repeat units<br>-Allele 2 ≥250 GAA repeat units	The diagnosis of GAA-*FGF14* ataxia (SCA27B) is confirmed. The report should mention the indication of genetic counselling for the family of the patient (offering confirmatory carrier testing)	The patient is a carrier of a *FGF14* GAA repeat expansion
